# Author Correction: Enhancement of trans-cleavage activity of Cas12a with engineered crRNA enables amplified nucleic acid detection

**DOI:** 10.1038/s41467-020-20117-z

**Published:** 2020-11-24

**Authors:** Long T. Nguyen, Brianna M. Smith, Piyush K. Jain

**Affiliations:** 1 Department of Chemical Engineering, University of Florida, 1006 Center Drive, Gainesville, FL 32611 USA; 2 UF Health Cancer Center, University of Florida, 2033 Mowry Road, CGRC 463, Gainesville, FL 32608 USA

**Keywords:** CRISPR-Cas9 genome editing, Assay systems, Molecular engineering

Correction to: *Nature Communications* 10.1038/s41467-020-18615-1, published online 30 September 2020.

In the original version of this Article, the composition of buffer D in the “Methods” section “LbCas12a expression and purification” was incorrectly provided as “(100 mM NaCl, 20 mM HEPES, 0.5 mM TCEP, pH = 8)”. It should have read “(2 M NaCl, 20 mM HEPES, 0.5 mM TCEP, pH = 8)”.

This has been corrected in both the PDF and HTML versions of this Article.

