# Supplementary material for: Enhancement of trans-cleavage activity of Cas12a with engineered crRNA enables amplified nucleic acid detection
Source: Nat Commun. 2020 Sep 30;11:4906. doi: 10.1038/s41467-020-18615-1 (PMC7528031; doi:10.1038/s41467-020-18615-1)
Supplement: Supplementary file 1 — Supplementary Information [file 41467_2020_18615_MOESM1_ESM.pdf]

Supplementary Information

Enhancement of trans-cleavage activity of Cas12a with engineered crRNA enables amplified nucleic acid detection

Long T. Nguyen[1], Brianna M. Smith[1], Piyush K. Jain[1,2]

[1]Department of Chemical Engineering, University of Florida, 1006 Center Drive, Gainesville, FL 32611, USA
[2]UF Health Cancer Center, University of Florida, 2033 Mowry Rd., CGRC 463, Gainesville, FL 32608, USA

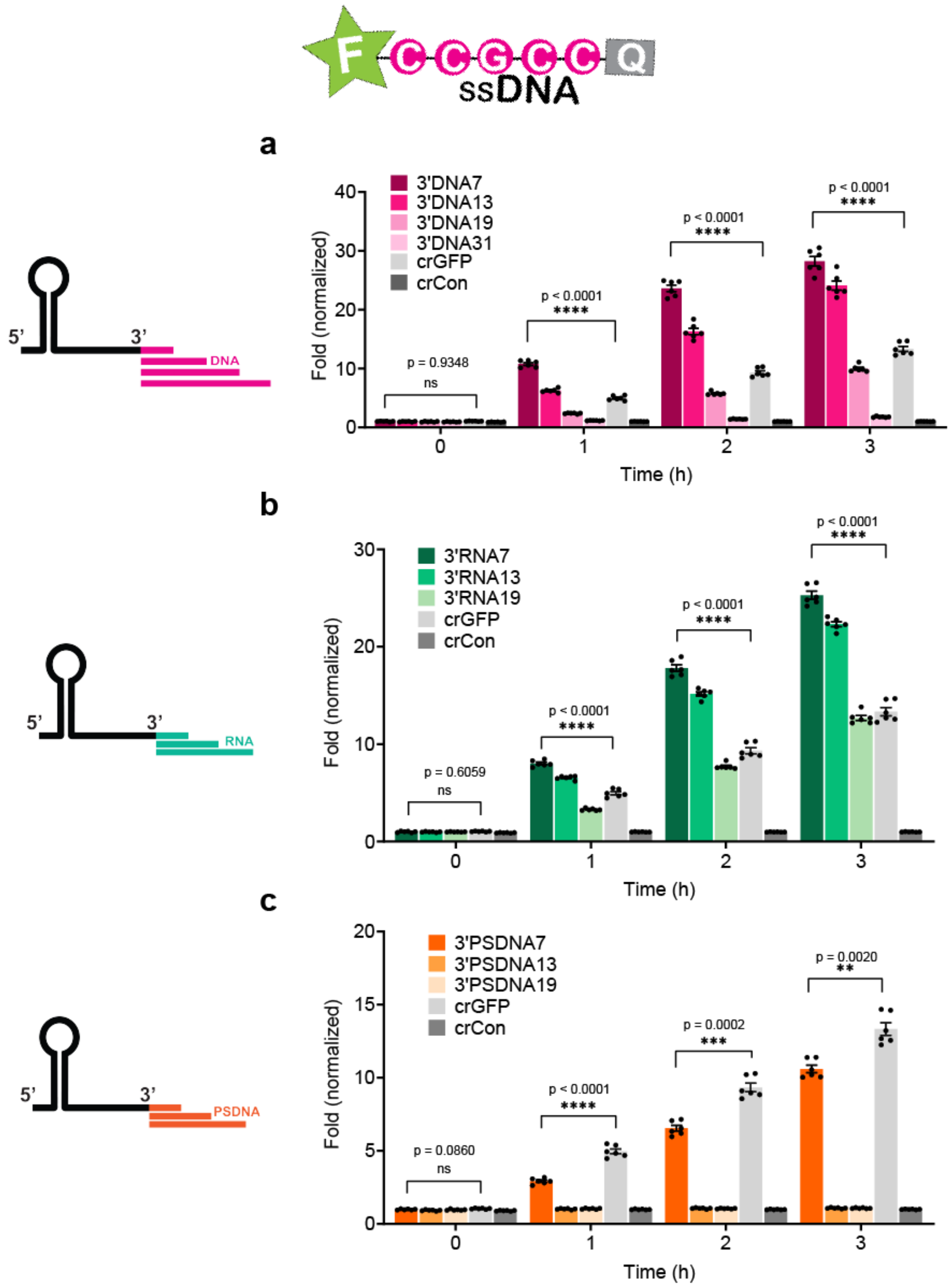

Supplementary Figure 1. Trans-cleavage activity of LbCas12a with modified 3' crRNA via fluorescence-quencher reporter assay with FAM-GC, where FAM-GC is a reporter shown above**.** F stands for fluorophore, and Q stands for quencher. (**a**) DNA, (**b**) RNA, and (**c**) PSDNA extensions of crRNA. The fold in fluorescence was normalized by taking the ratio of background-corrected fluorescence signals of the samples with the activator to the corresponding samples without the activator. Error bars in (a), (b), and (c) represent mean ± SEM, where n = 6 replicates (three technical replicates examined over two independent experiments). Statistical analysis was performed using two-way ANOVA test with Dunnett's multiple comparison test, where ns = not significant, and the asterisk (*) denotes p values.

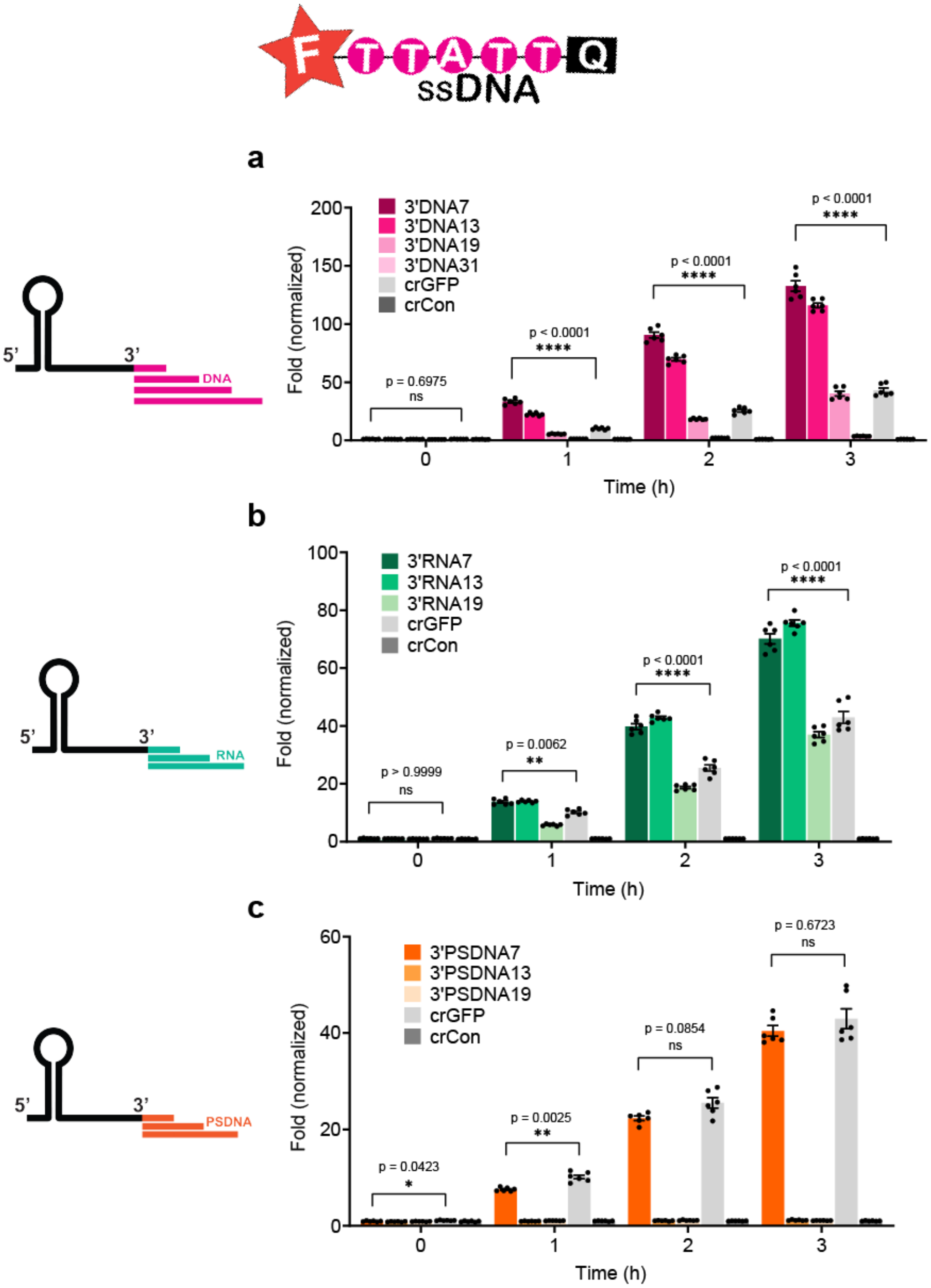

Supplementary Figure 2. Trans-cleavage activity of LbCas12a with modified 3’ crRNA via fluorescence-quencher reporter assay with HEX-TA, where HEX-TA is a reporter shown above. F stands for fluorophore, and Q stands for quencher. (**a**) DNA, (**b**) RNA, and (**c**) PSDNA extensions of crRNA. The fold in fluorescence was normalized by taking the ratio of background-corrected fluorescence signals of the samples with the activator to the corresponding samples without the activator. Error bars in (a), (b), and (c) represent mean ± SEM, where n = 6 replicates (three technical replicates examined over two independent experiments). Statistical analysis was performed using two-way ANOVA test with Dunnett’s multiple comparison test, where ns = not significant, and the asterisk (*) denotes p values.

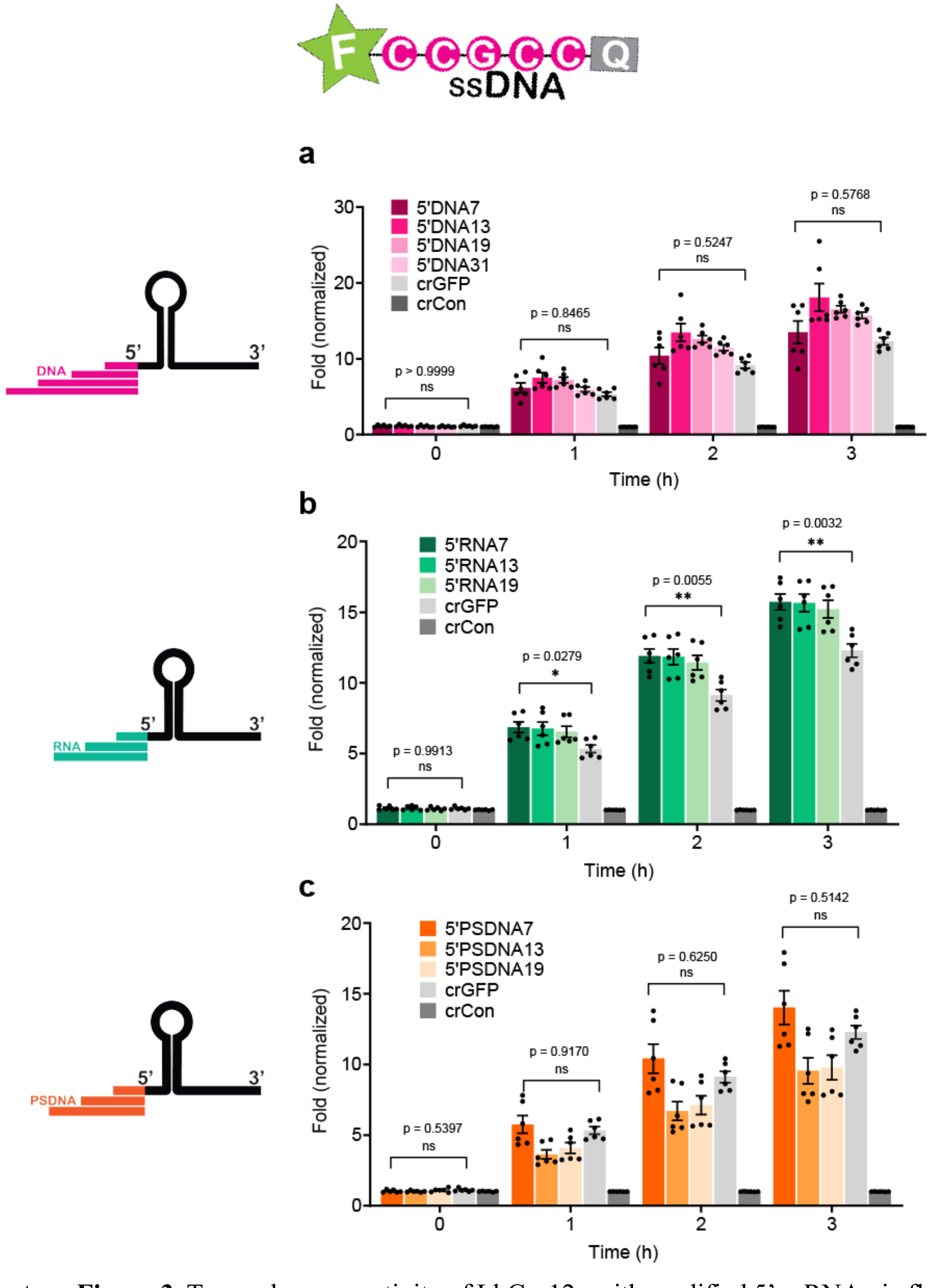

Supplementary Figure 3. Trans-cleavage activity of LbCas12a with modified 5' crRNA via fluorescence-quencher reporter assay with FAM-GC, where FAM-GC is a reporter shown above. F stands for fluorophore, and Q stands for quencher. (**a**) DNA, (**b**) RNA, and (**c**) PSDNA extensions of crRNA. The fold in fluorescence was normalized by taking the ratio of background-corrected fluorescence signals of the samples with the activator to the corresponding samples without the activator. Error bars in (a), (b), and (c) represent mean ± SEM, where n = 6 replicates (three technical replicates examined over two independent experiments). Statistical analysis was performed using two-way ANOVA test with Dunnett's multiple comparison test, where ns = not significant, and the asterisk (*) denotes p values.

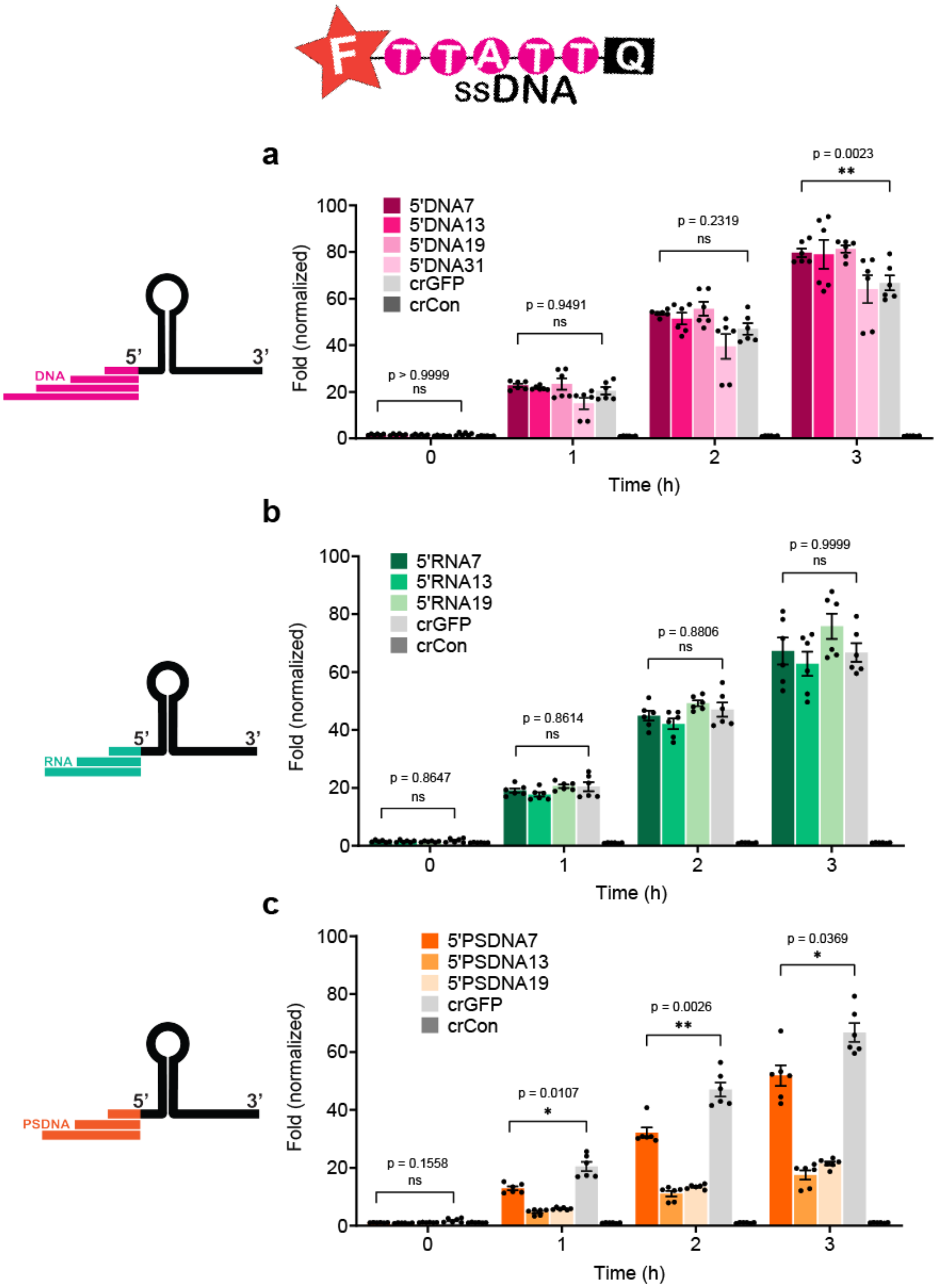

Supplementary Figure 4. Trans-cleavage activity of LbCas12a with modified 5' crRNA via fluorescence-quencher reporter assay with HEX-TA, where HEX-TA is a reporter shown above. F stands for fluorophore, and Q stands for quencher. (**a**) DNA, (**b**) RNA, and (**c**) PSDNA extensions of crRNA. The fold in fluorescence was normalized by taking the ratio of background-corrected fluorescence signals of the samples with the activator to the corresponding samples without the activator. Error bars in (a), (b), and (c) represent mean ± SEM, where n = 6 replicates (three technical replicates examined over two independent experiments). Statistical analysis was performed using two-way ANOVA test with Dunnett's multiple comparison test, where ns = not significant, and the asterisk (*) denotes p values.

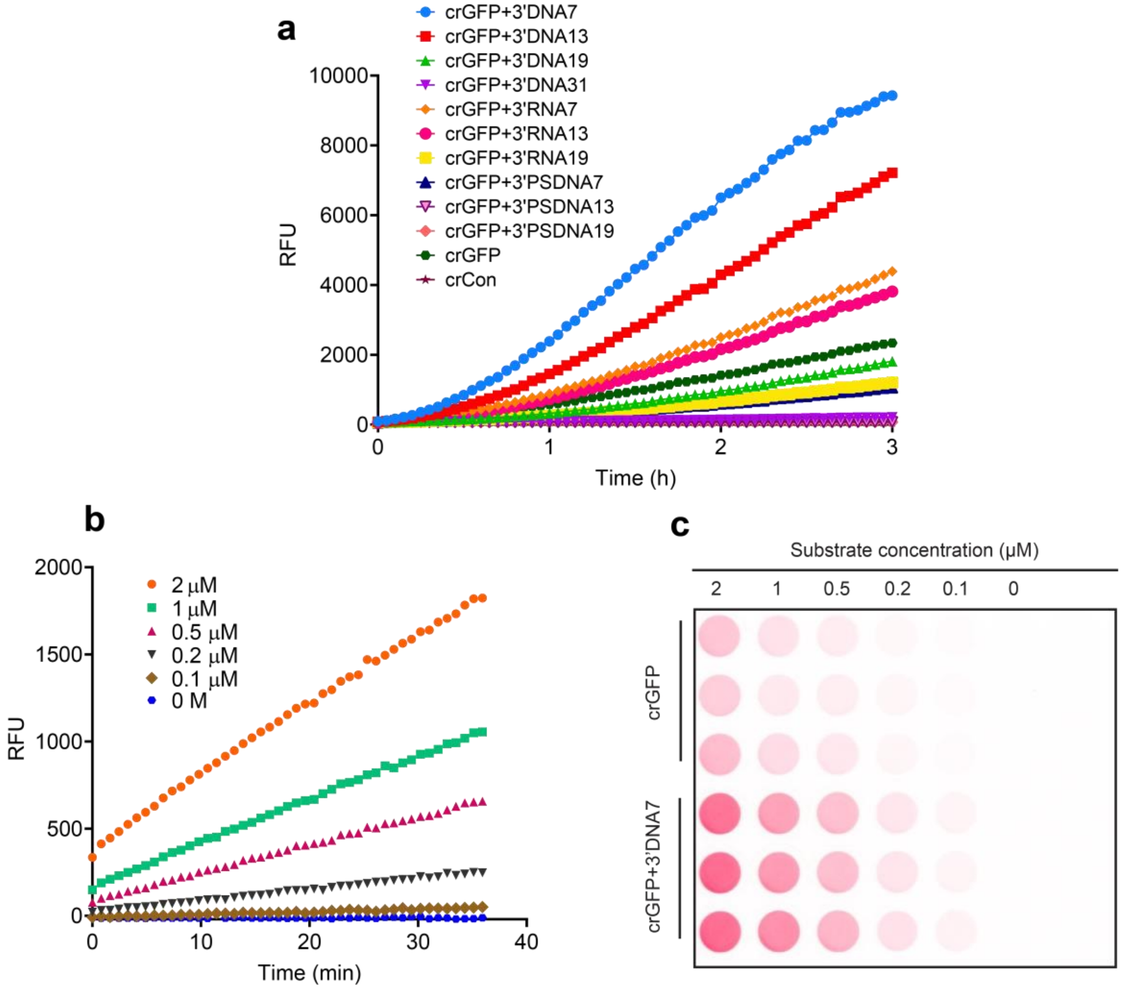

Supplementary Figure 5. Representatives of samples from the fluorescence-quencher reporter assay. (**a**) Fluorescence measurement in relative fluorescence unit (RFU) of LbCas12a reaction with 3'-end modified crGFP. (**b**) Fluorescence reading of the Michaelis-Menten study. The substrate used in this experiment was /5HEX/TTATT/3IABkFQ/. (**c**) Fluorescence image of (b) scanned by Amersham Typhoon.

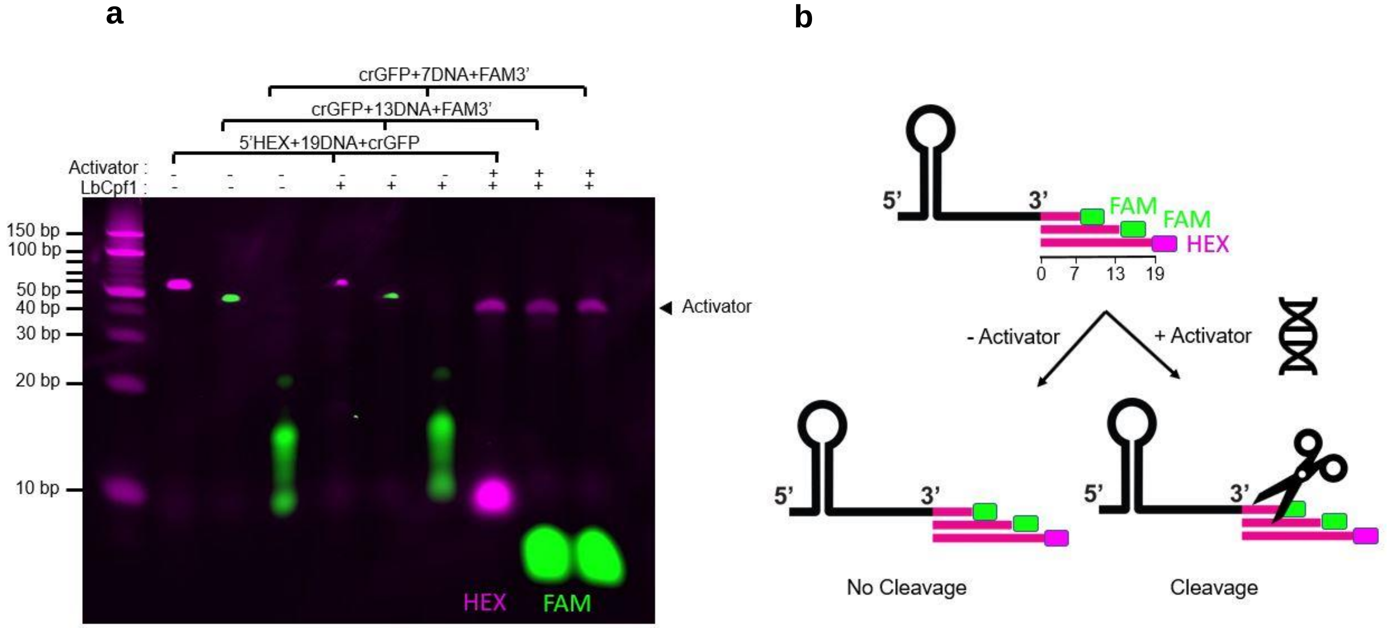

Supplementary Figure 6. Cis-cleavage of LbCas12a with fluorescently-labeled extended crGFP. (**a**) The 3'- end extensions of crGFP were varied from 0 to 19-mer DNA and tagged with FAM/HEX. (**b**) The proposed mechanism for LbCas12a processing of modified crGFP as observed from (a). The experiment in (a) was repeated three times with similar results.

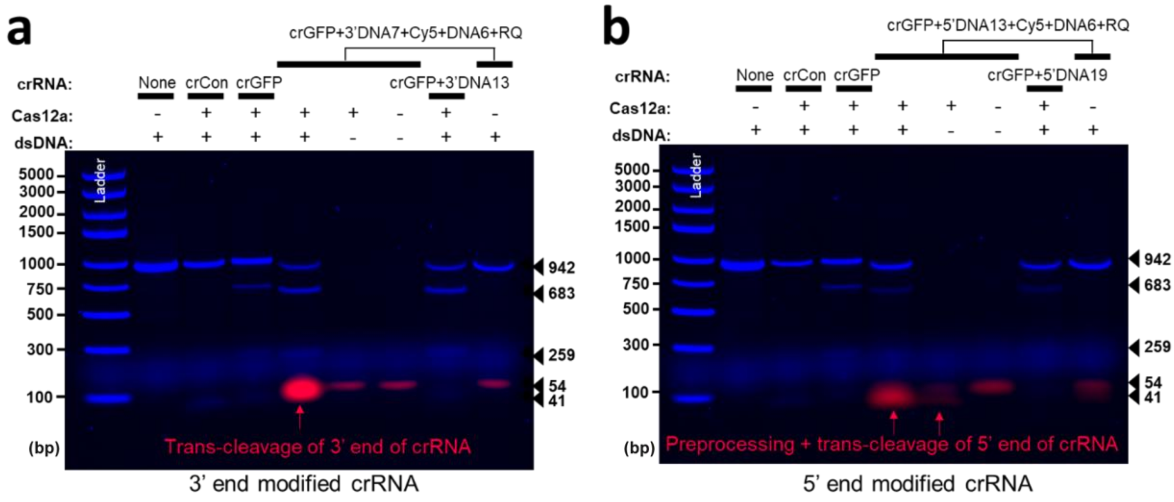

Supplementary Figure 7. Gel images of cis-cleavage assay of LbCas12a with different crRNAs carrying a fluorophore-quencher pair on either (a) 3' end or (b) 5' end. crCon (scrambled crRNA), crGFP (GFP targeting crRNA), (a) crGFP+3' DNA13, and crGFP+3' DNA7+Cy5+DNA6+Iowa Black RQ or (b) 5'-end modified crRNAs including crGFP+5' DNA19 and crGFP+5' DNA13+Cy5+DNA6+Iowa Black RQ. Cy5 is indicated in red and DNA stained with GelRed is shown in blue. The 5' end modified crRNAs showed cleavage of crRNA immediately after adding the LbCas12a but before adding the activator. However, both 3' and 5' end modified crRNAs, showed increase in signal intensity after activator addition indicating trans cleavage of the crRNA. 250 nM of LbCas12a, 250 nM of crRNA, and 7.4 nM of DNA activator fragment were used. The experiments in (a) and (b) were repeated twice with similar results.

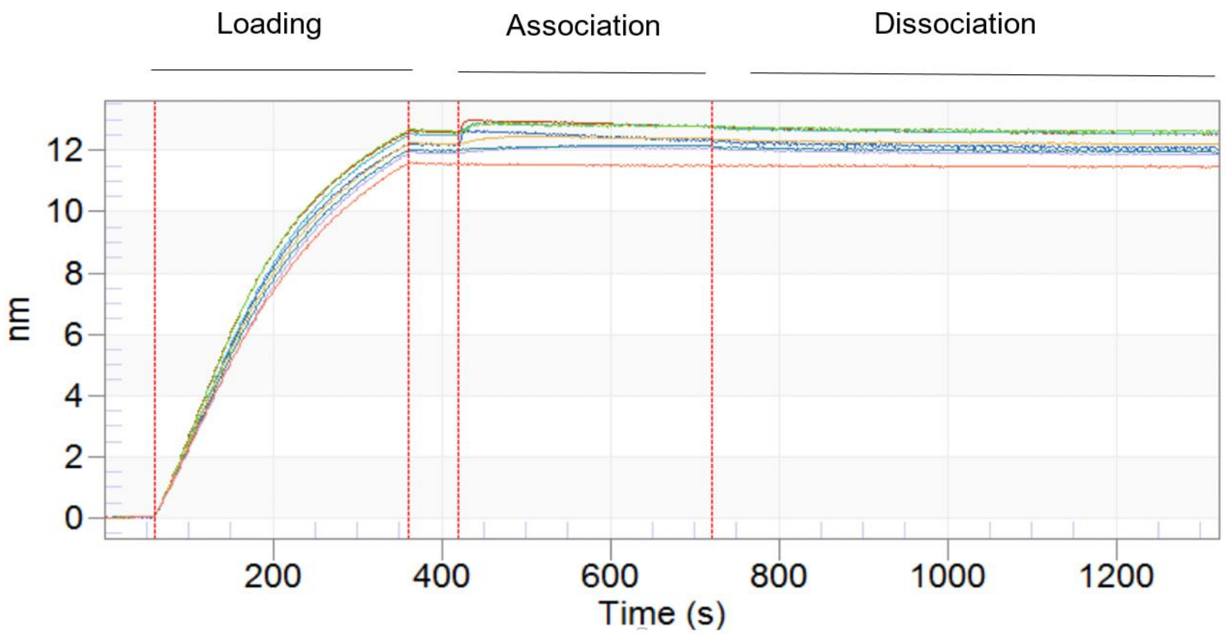

Supplementary Figure 8. A representation of the biolayer interferometry (BLI) binding kinetics. The picture shown above is the binding kinetic study of LbCas12a to crGFP+3'DNA7. The experiment was carried in five steps: baseline1, loading, baseline2, association, and dissociation (see materials and methods). The y-axis represents response of LbCas12a and crRNA to the biosensor in nm. Data were trimmed and processed using the Octet Data Analysis 10.0 software, and only K_D values with $R^2 > 0.9$ were selected.

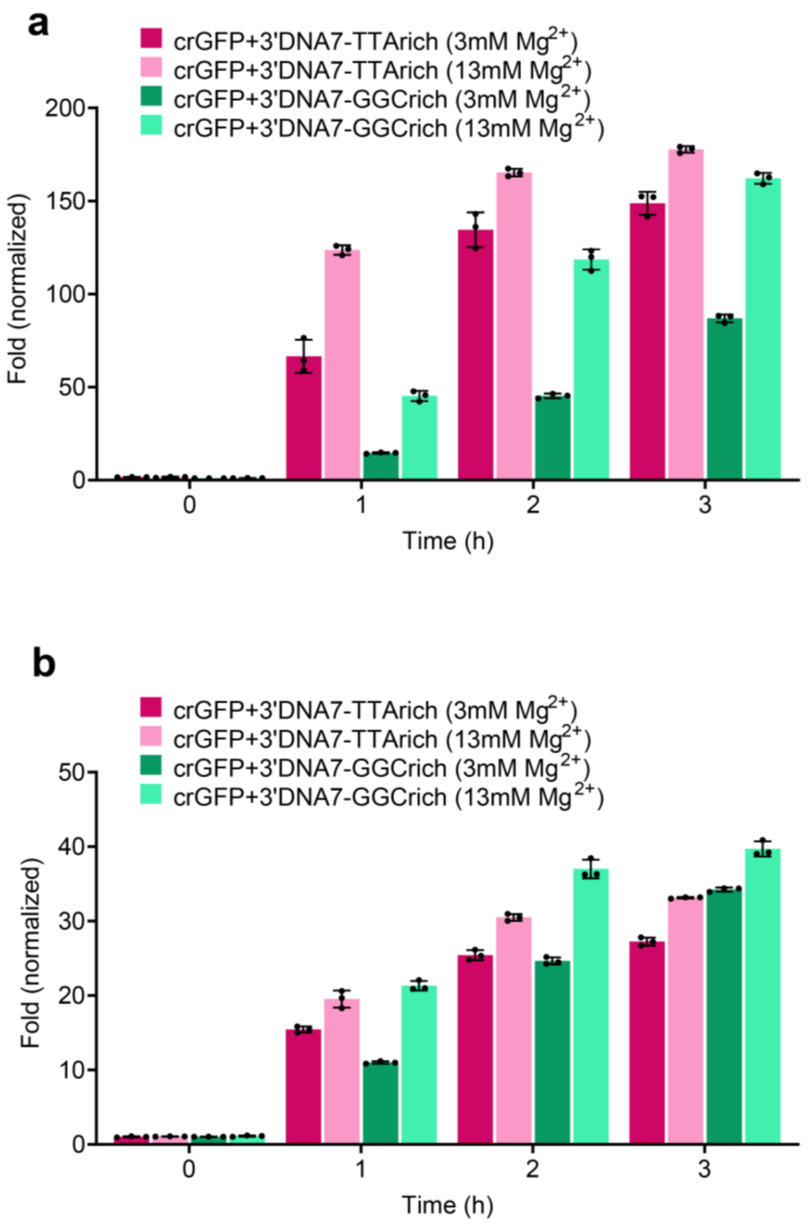

Supplementary Figure 9. Trans-cleavage activity of LbCas12a with modified crGFP+3'DNA7 with either GC rich or TA rich region via fluorescence-quencher reporter assay at varying Mg^{2+} concentration**.** F stands for fluorophore, and Q stands for quencher. (**a**) FAM-TA was used, (**b**) FAM-GC was used. The fold in fluorescence was normalized by taking the ratio of background-corrected fluorescence signals of the samples with the activator to the corresponding samples without the activator. Error bars represent mean ± SD, where n = 3 technical replicates.

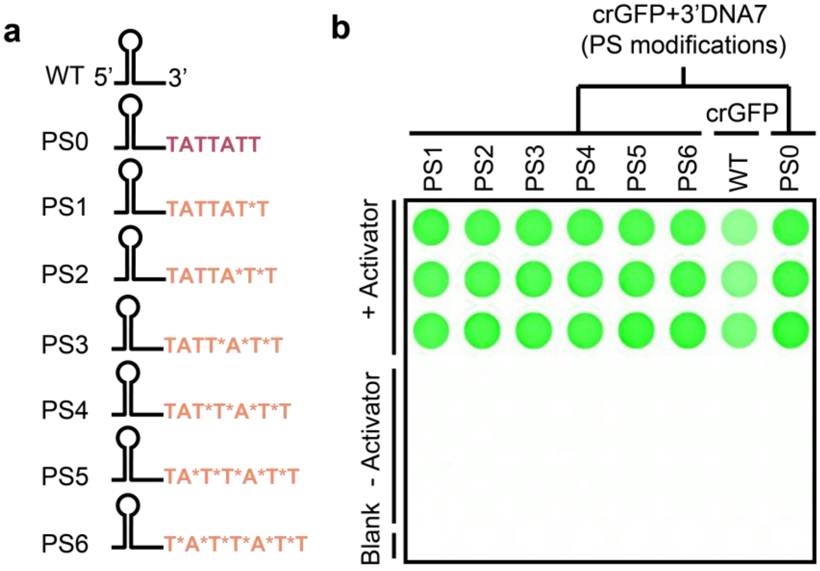

Supplementary Figure 10. Typhoon image (scanned with Amersham Typhoon, GE Healthcare) of the LbCas12a fluorescence-based reporter assay in Figure 2c,d in the main text.

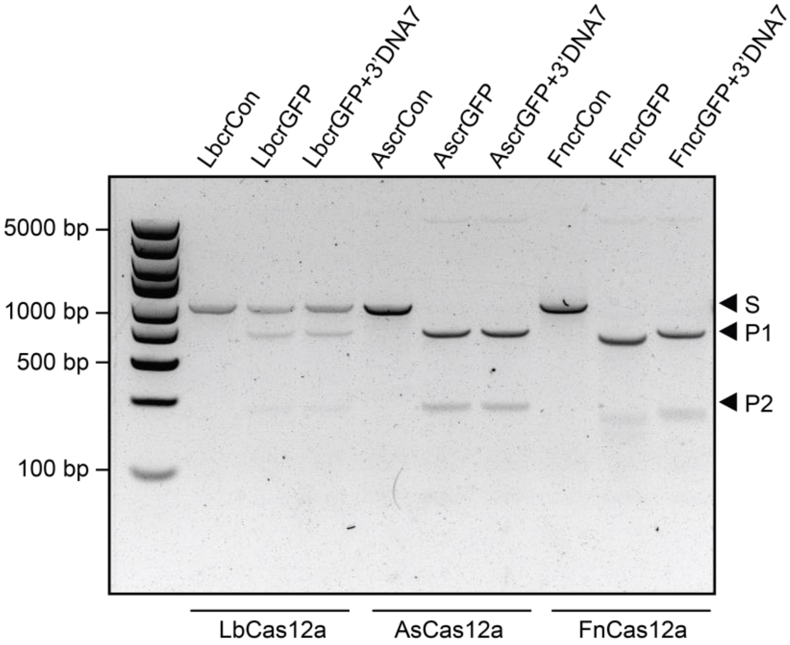

Supplementary Figure 11. Cis-cleavage and trans-cleavage activity of LbCas12 with modified crGFP and crGFP+3'DNA7 with LbCas12a, AsCas12a, and FnCas12a**.** The cis-cleavage reaction was loaded on 1% native agarose gel. The experiment was repeated twice with similar results.

a

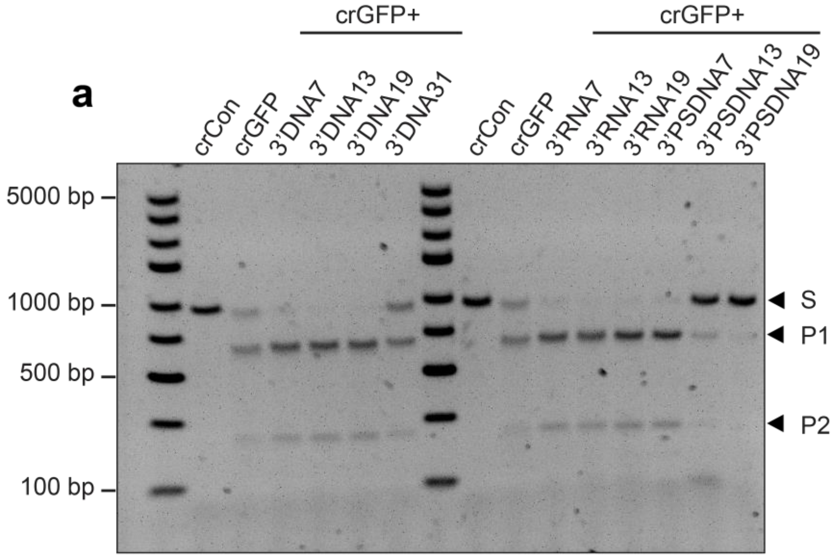

b

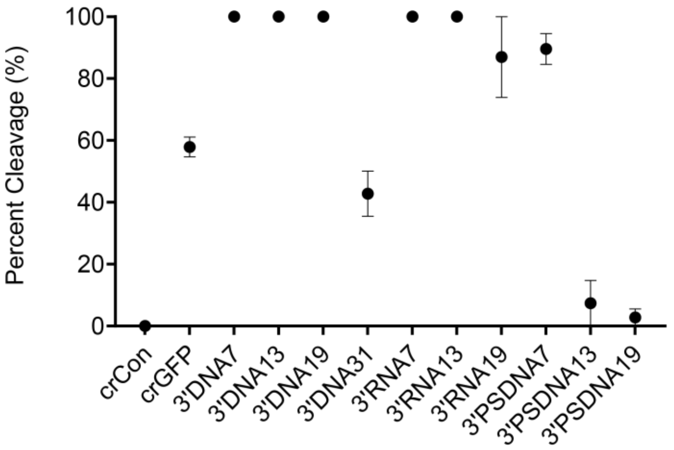

Supplementary Figure 12. Cis-cleavage of LbCas12a with 3'-end modified crGFP. For this assay, 100nM of LbCas12a, 100 nM of crRNA, and 7.4 nM of GFP fragment were used. crCon represents nonspecific crRNA. (**a**) 1% agarose gel image. (**b**) Percent cleavage of the GFP fragment calculated in (**a**), where error bars represent mean ± SD with n = 2 replicates. The experiment was repeated more than twice with similar results.

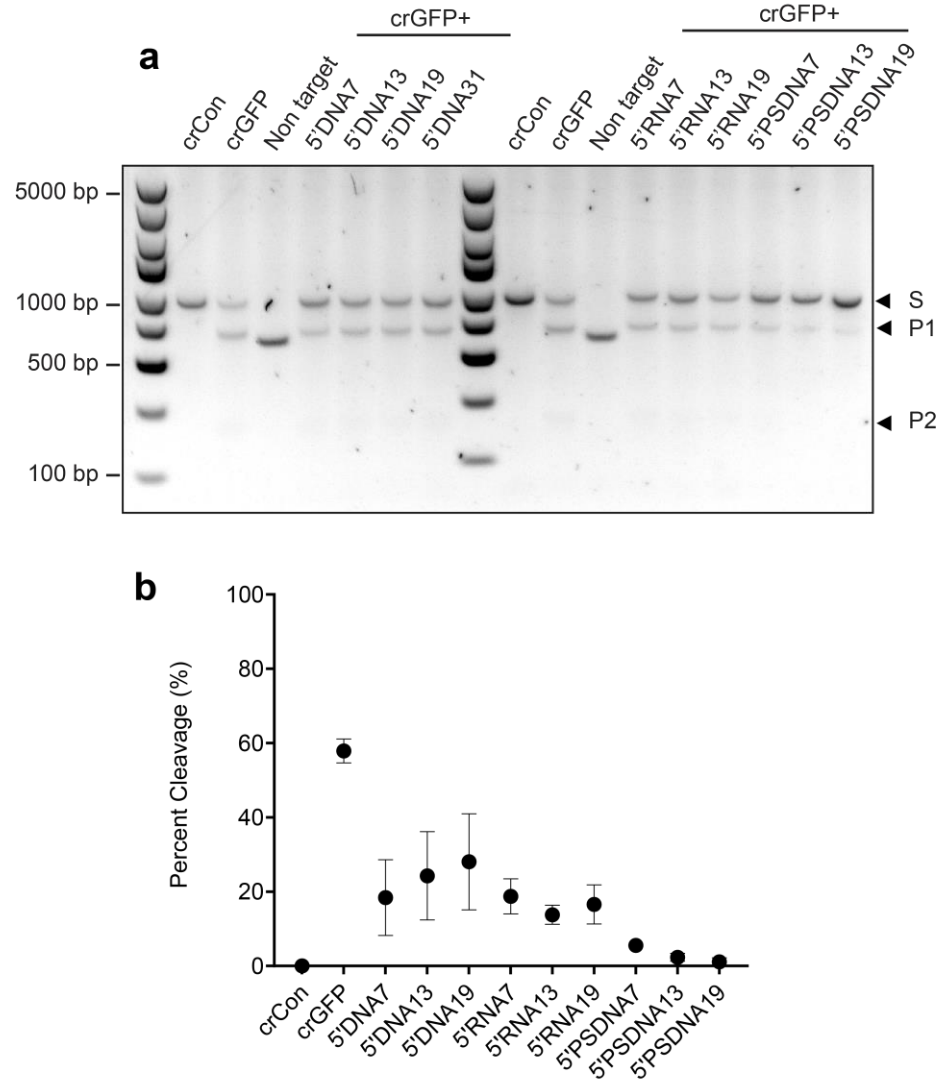

Supplementary Figure 13. Cis-cleavage of LbCas12a with 5'-end modified crGFP. For this assay, 100nM of LbCas12a, 100 nM of crRNA, and 7.4 nM of GFP fragment were used. crCon represents nonspecific crRNA. (**a**) 1% agarose gel image. (**b**) Percent cleavage of the GFP fragment calculated in (**a**), where error bars represent mean ± SD with n = 2 replicates. The experiment was repeated more than twice with similar results.

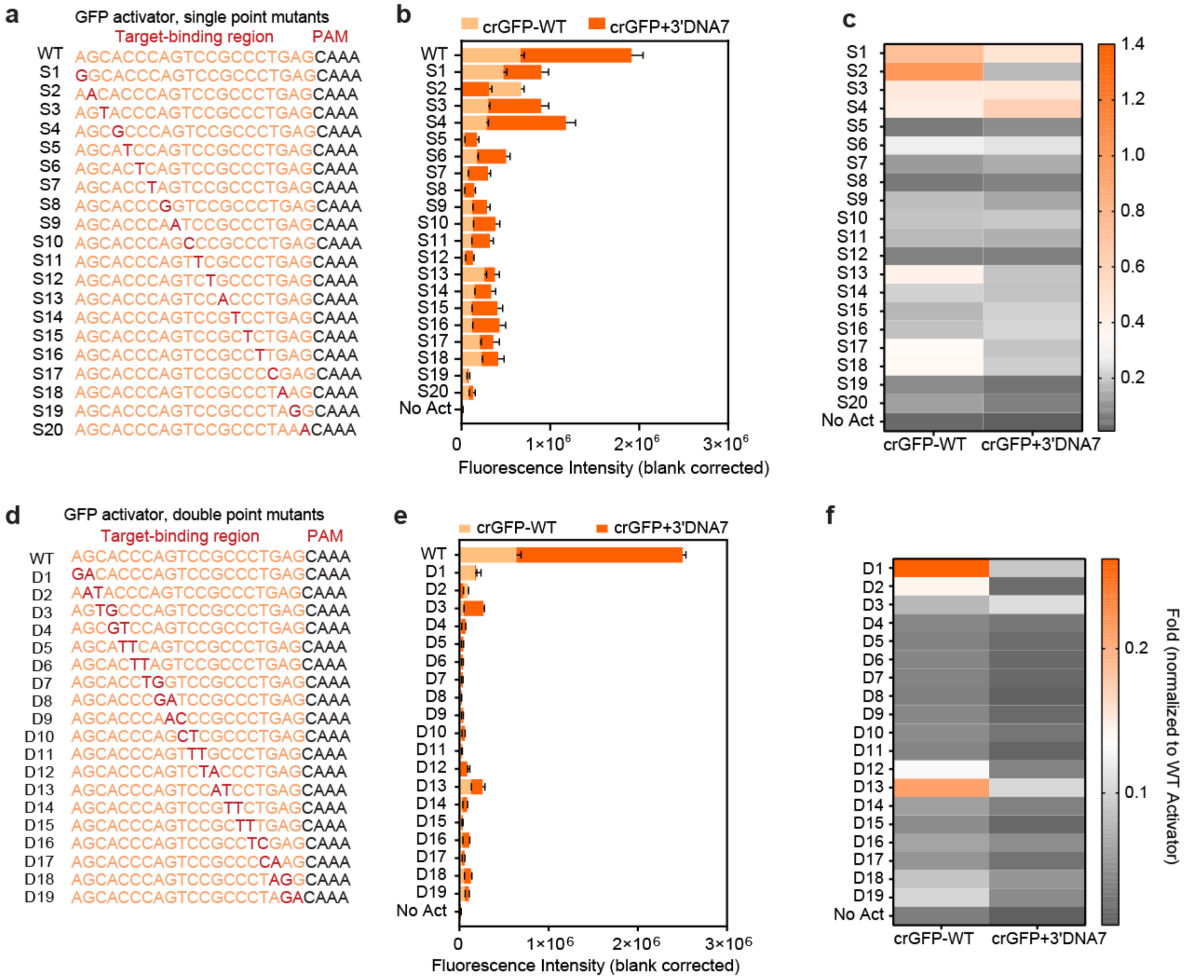

Supplementary Figure 14. Effect of single-point and double-point mutations on the target strand of the double-stranded GFP fragment demonstrated as raw fluorescence signals of Figure 4a-d. (**a**) Sequence representation of dsDNA GFP WT and single-point mutants. The heat map of (**b**) the raw fluorescence intensity and (**c**) the normalized fluorescence intensity with respect to the wild-type activator. Error bars in (b) and (c) represent mean ± SEM, where n = 6 replicates (three technical replicates examined over two independent experiments). (**d**) Sequence representation of dsDNA GFP WT and double-point mutants. (**e**) Raw fluorescence intensity and (**f**) the normalized fluorescence intensity with respect to the wild-type activator for 19 double-point mutation of target GFP fragments. Error bars in (d) and (e) represent mean ± SEM, where n = 4 replicates (two technical replicates examined over two independent experiments). The same data in (c) and (f) was plotted as a bar graph with error bars the Figure 4b and Figure 4d, respectively.

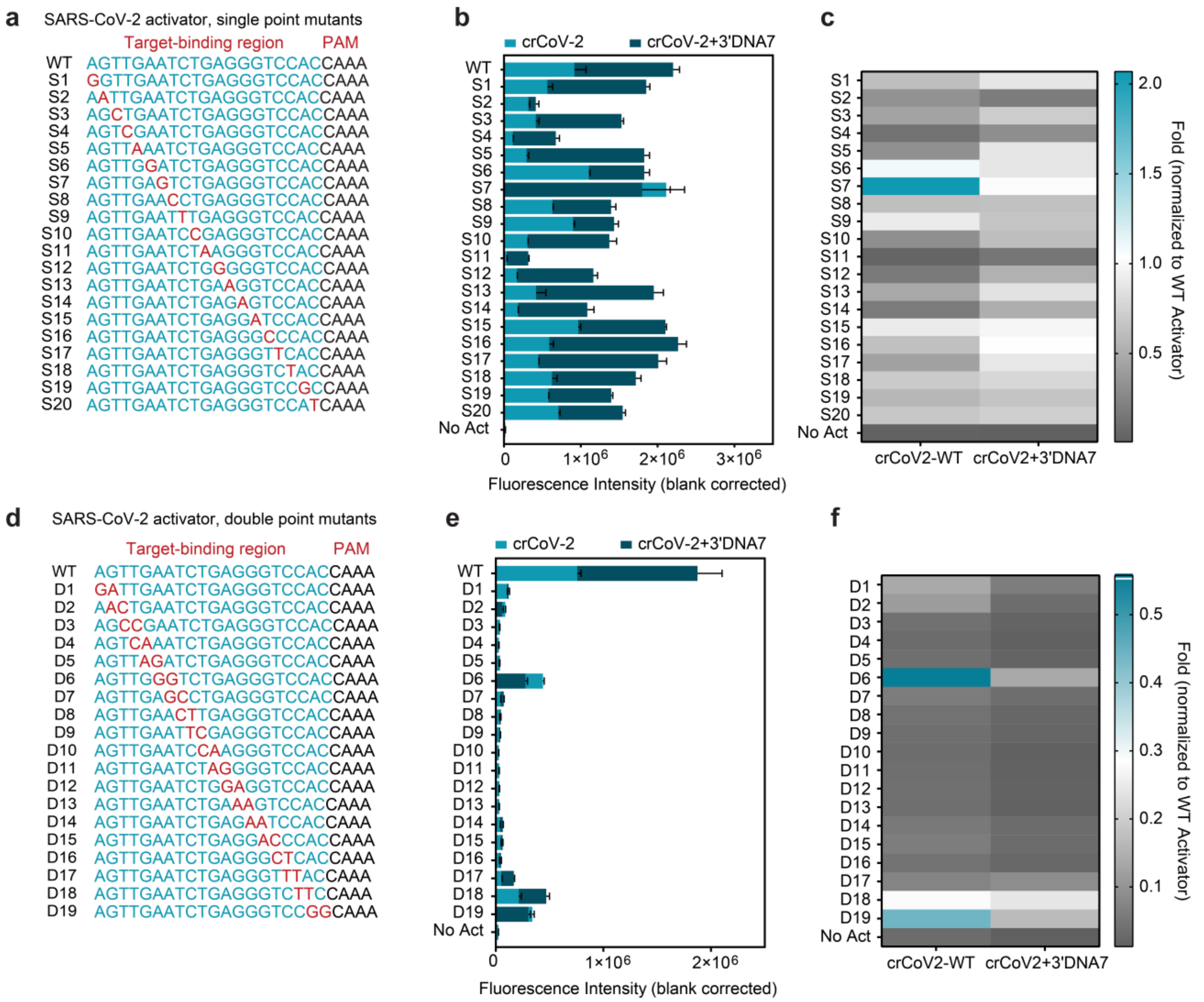

Supplementary Figure 15. Effect of single-point and double-point mutations on the target strand of the double-stranded DNA SARS-CoV-2 demonstrated as raw fluorescence signals of Figure 4e-h. (**a**) Sequence representation of dsDNA SARS-CoV-2 WT and single-point mutants. (**b**) Raw fluorescence intensity and (**c**) the normalized fluorescence intensity with respect to the wild-type activator, for 20 single-point mutants of the target SARS-CoV-2. (**d**) Sequence representation of dsDNA SARS-CoV-2 WT and double-point mutants. (**e**) Raw fluorescence intensity and (**f**) the normalized fluorescence intensity with respect to the wild-type activator, after 20 minutes for 19 double-point mutants of the target. Error bars in (b), (c), (d) and (e) represent mean ± SEM, where n = 4 replicates (two technical replicates examined over two independent experiments). The same data in (c) and (f) were plotted as bar graph with error bars in the Figure 4f and Figure 4h, respectively.

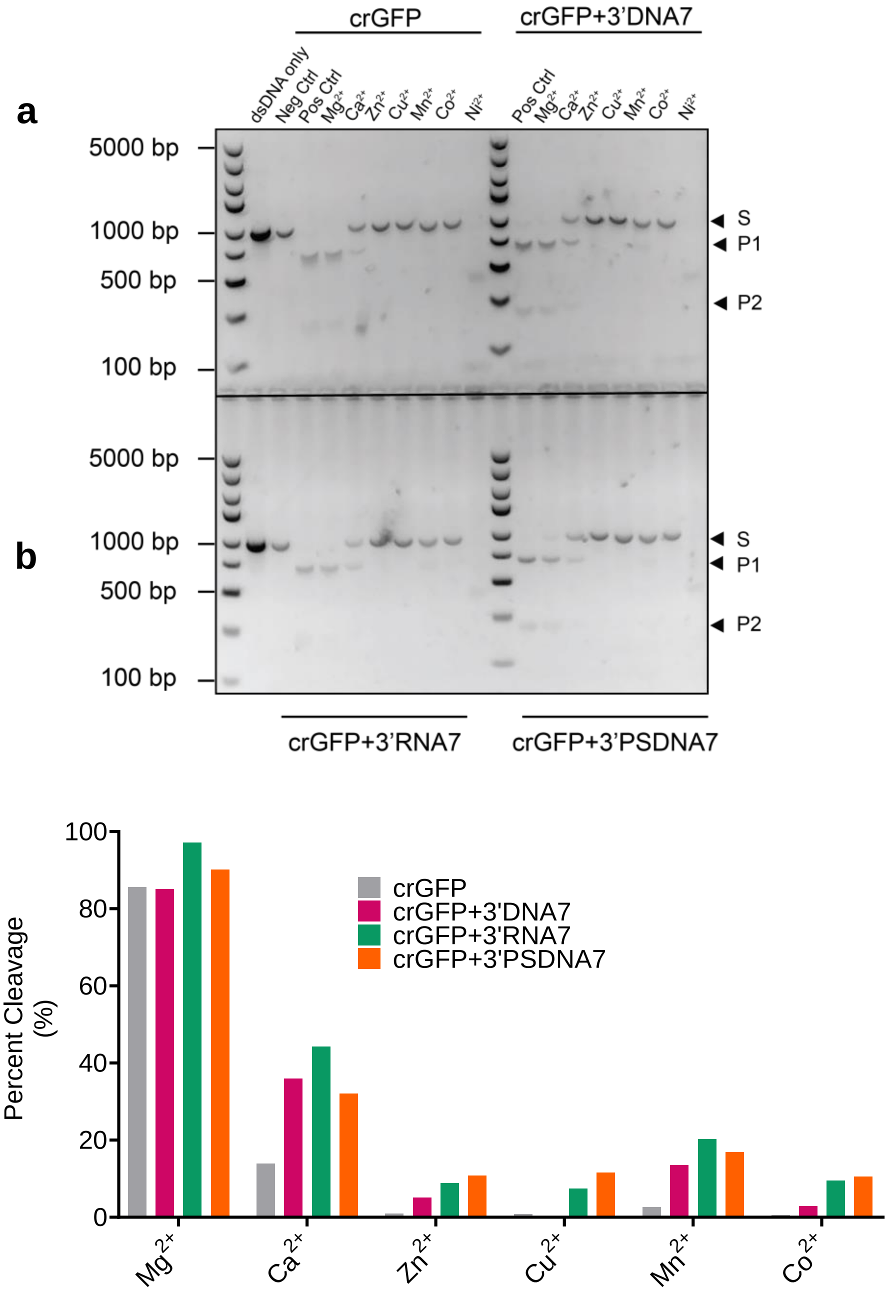

Supplementary Figure 16. (a) the effect of a divalent ion on the cis-cleavage assay of LbCas12a with different crRNAs including wild-type crGFP (top-left quadrant), crGFP+3'DNA7 (top-right quadrant), crGFP+3'RNA7 (bottom-left quadrant), and crGFP+3'PSDNA7 (bottom-right quadrant). Neg Ctrl represents the negative control where crCon was used in the reaction mixture. Pos Ctrl represents the positive control where NEBuffer 2.1 was added. In this experiment, 100 nM of LbCas12a, 100 nM of crRNA, and 6.6 nM of DNA activator fragment were used. (**b**) Percent cleavage analyzed in (a). Each metal ion was added to LbCas12a reaction to a final concentration of 3mM in cleavage buffer (50 mM NaCl, 10 mM Tris-HCl, 10 mM $MgCl_2$, 100 µg/ml BSA, pH 7.9). The graph indicated mean from a pilot experiment in the graph (n=1) that was not repeated. Only Mg^{2+} and Zn^{2+} were further studied (repeated twice with similar results).

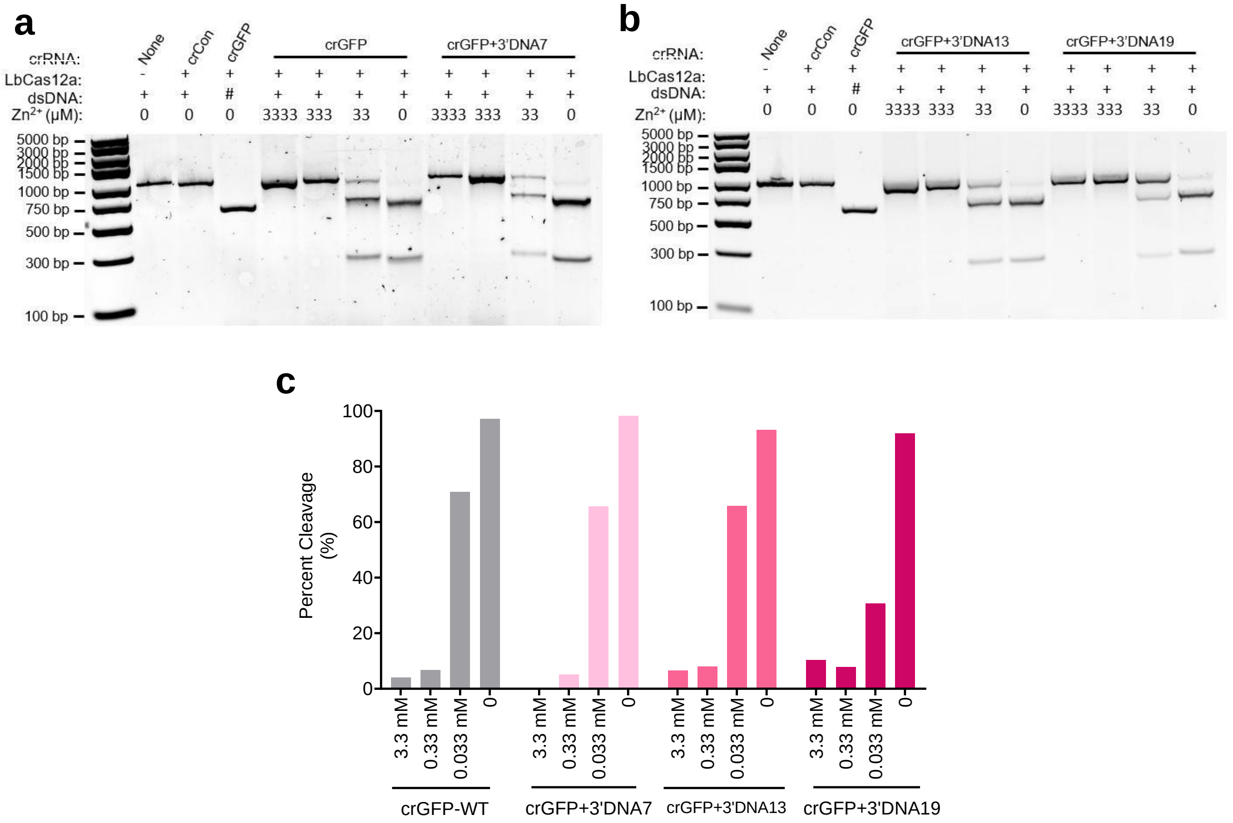

Supplementary Figure 17. The effect of Zn^{2+} on Cis-cleavage assay of LbCas12a with different crRNAs in the presence of Mg^{2+}. 200 nM of LbCas12a, 200 nM of crRNA, 6.6 nM of DNA activator fragment, and 3mM of Mg^{2+} were used. # stands for non-target dsDNA fragment. (**a**) crGFP and crGFP+3'DNA7. (**b**) crGFP+3'DNA13 and crGFP+3'DNA19. (**c**) Percent cleavage in (a) and (b). Zn^{2+} ion concentration was serial diluted and added to the LbCas12a reaction in the presence cleavage buffer (NEBuffer 2.1 with 3mM Mg^{2+} final concentration. The experiments in (a) and (b) were repeated once.

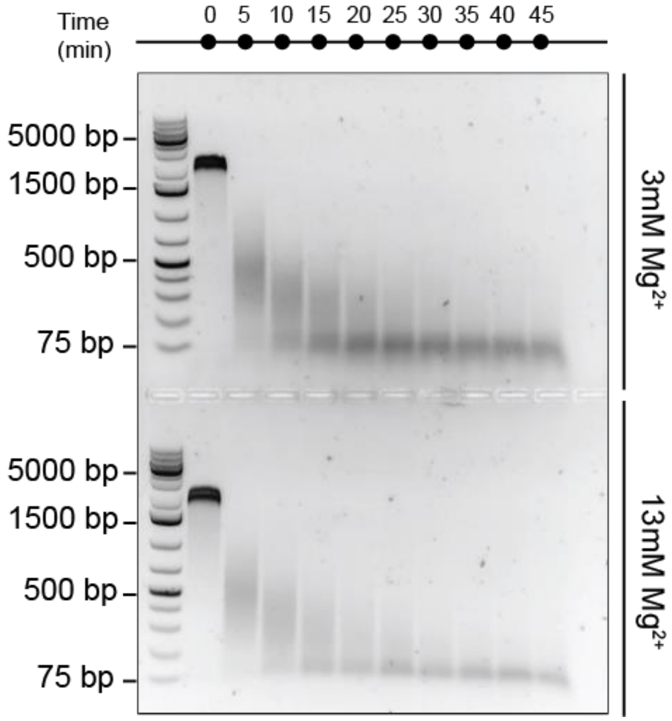

Supplementary Figure 18. Time-dependent cis-cleavage of LbCas12a on GFP in the presence of nonspecific ssDNA M13mp18 at varying Mg^{2+} concentration. The reaction mixture was taken out every five minutes and quenched with 6X SDS-containing loading dye.

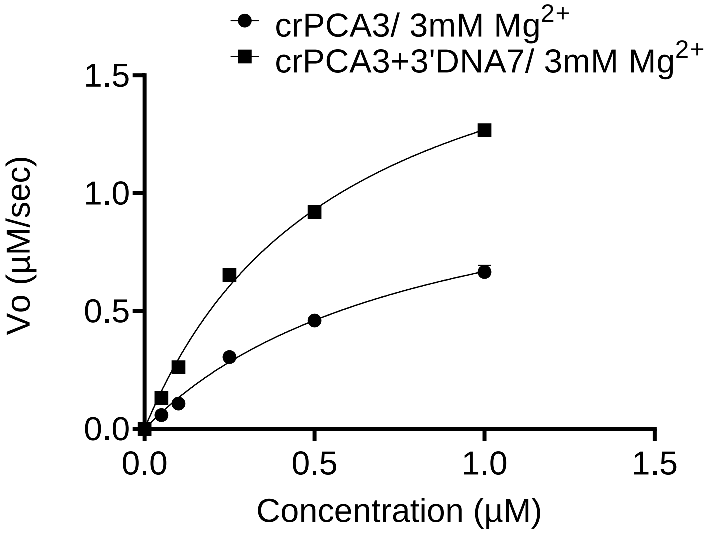

Supplementary Figure 19. Michaelis-Menten kinetic study of the wild type crPCA3 vs. crPCA3+3'DNA7. The graph shows initial velocity as a function of substrate concentration. In this case, the substrate used was /5HEX/TTATT/3IABkFQ/.

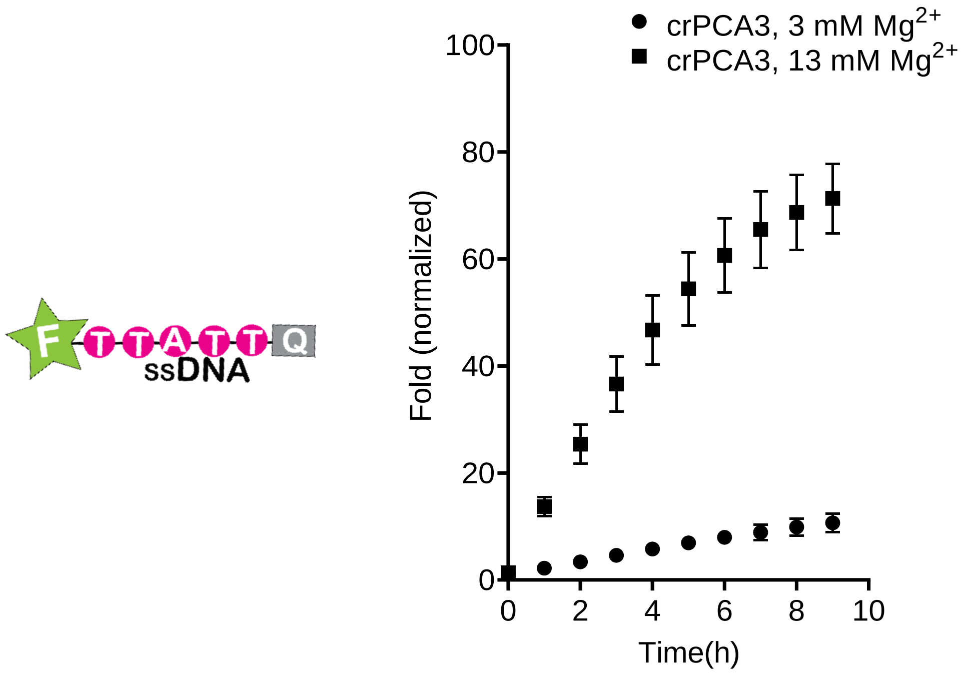

Supplementary Figure 20. Trans-cleavage activity of LbCas12 with modified 3' crPCA3 via fluorescence-quencher reporter assay FAM-TA at varying Mg^{2+} concentration. FAM-TA is a reporter shown above. F stands for fluorophore, and Q stands for quencher. The fold in fluorescence was normalized by taking the ratio of background-corrected fluorescence signals of the samples with the activator to the corresponding samples without the activator. Error bars represent mean ± SEM, where n = 6 replicates (three technical replicates examined over two independent experiments).

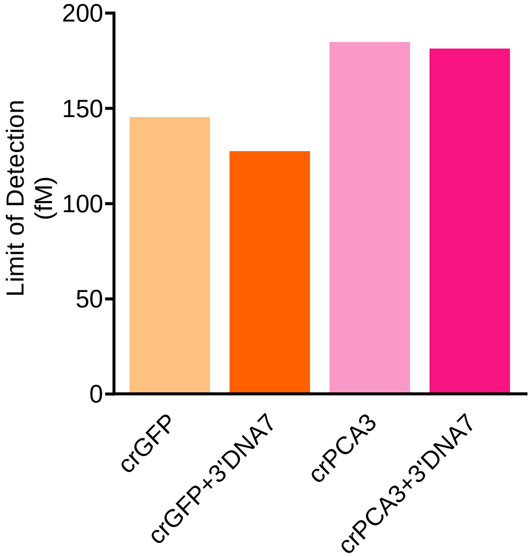

Supplementary Figure 21. Limit of detection using modified crRNA/LbCas12a system. Limit of detection in femtomolar at 13 mM Mg^{2+} concentration. The reaction was carried out by adding simulated human urine spiked with either dsDNA GFP or PCA3 fragments to the LbCas12a reaction mixture.

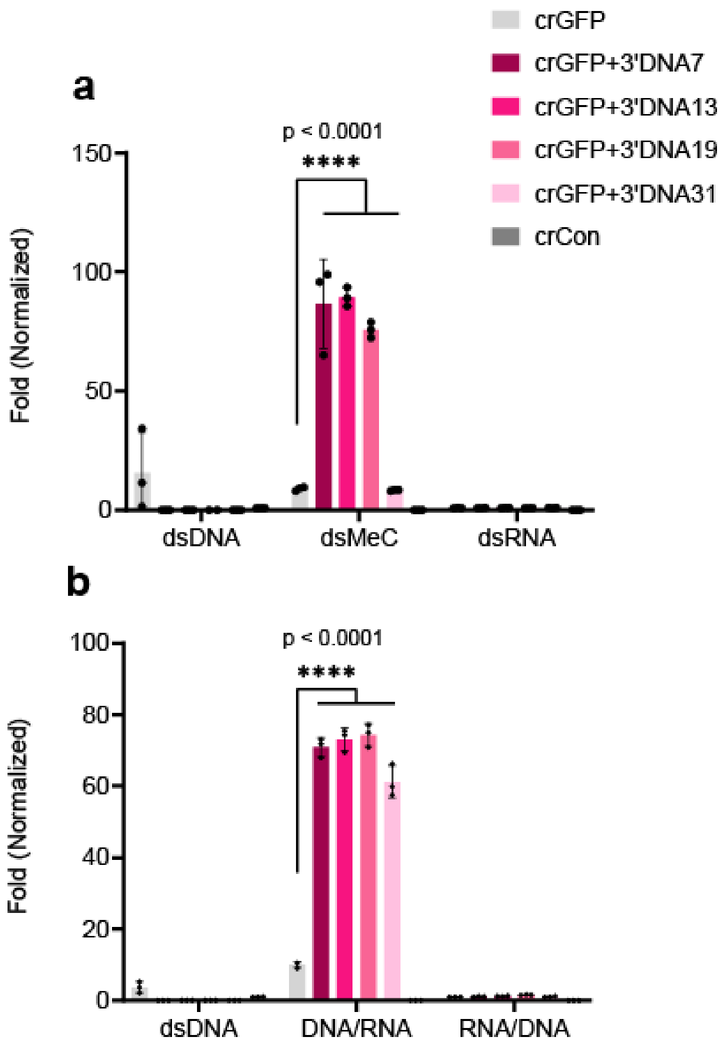

Supplementary Figure 22. LbCas12a trans-cleavage with different modified crRNAs and double-stranded activators (target and non-target strands annealed in the ratio of 1:1) measured by a fluorescence-quencher reporter assay (FAM-TA) in triplicates (n=3) . The data shows results at 81 minutes for (**a**) dsDNA, dsMeC, sRNA, and (**b**) dsDNA, DNA/RNA, RNA/DNA. The values were normalized to their respective crRNA without activators. The error bars in (a) and (b) denote mean ± SD, where n = 3 technical replicates. Statistical analysis was performed using two-way ANOVA test with Dunnett's multiple comparison test, where the asterisk (*) denotes p values.

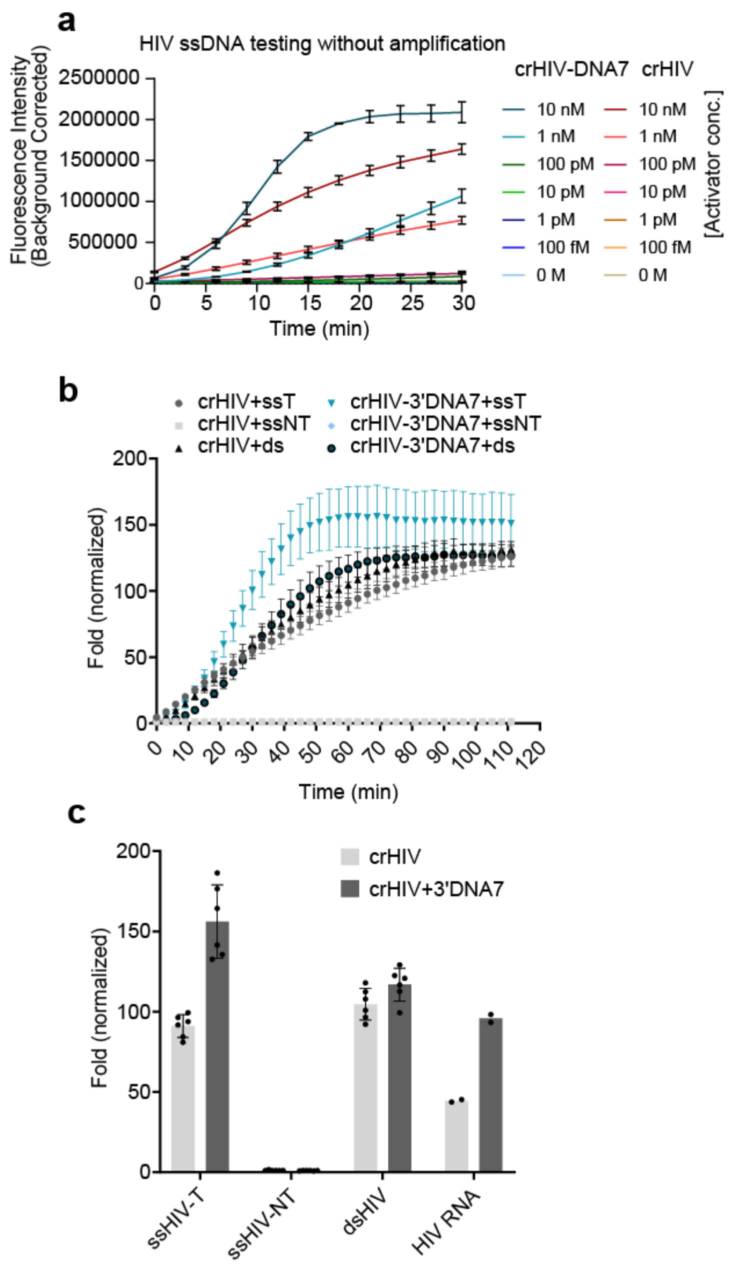

Supplementary Figure 23. (**a**) Trans-cleavage activity of regular crRNA vs. 3'DNA7 modified crRNA for detecting HIV ssDNA with LbCas12a over 30 minutes in the presence of various concentrations of HIV target dsDNA. Blank subtracted raw fluorescence intensities are indicated. Error bars represents mean ± SD, where n = 3 technical replicates. (**b**) Fold change in fluorescence intensity in the presence vs. absence of target is indicated for various HIV ssDNA vs. dsDNA activators, where ssT indicates ssDNA target strand, ssNT indicates ssDNA non-target strand and ds indicates double-stranded DNA target. Error bars represent mean ± SEM where n = 6 replicates (three technical replicates examined over two independent experiments). (**c**) Trans-cleavage of ssHIV using engineered CRISPR/LbCas12a. Comparison of single-stranded (ss) vs. double-stranded DNA (ds) targets analysis after 30 minutes is shown in bar graphs. HIV-1 ssDNA from Tat gene (IDT Technologies). The modified crRNA showed much higher sensitivity than he regular crRNA with fM detection limits within 30 minutes. For ssHIV-T, ssHIV-NT, and dsHIV, error bars represent mean ± SEM with n = 6 replicates (three technical replicates examined over two independent experiments). For HIV RNA, error bars represent mean with n = 2 replicates.

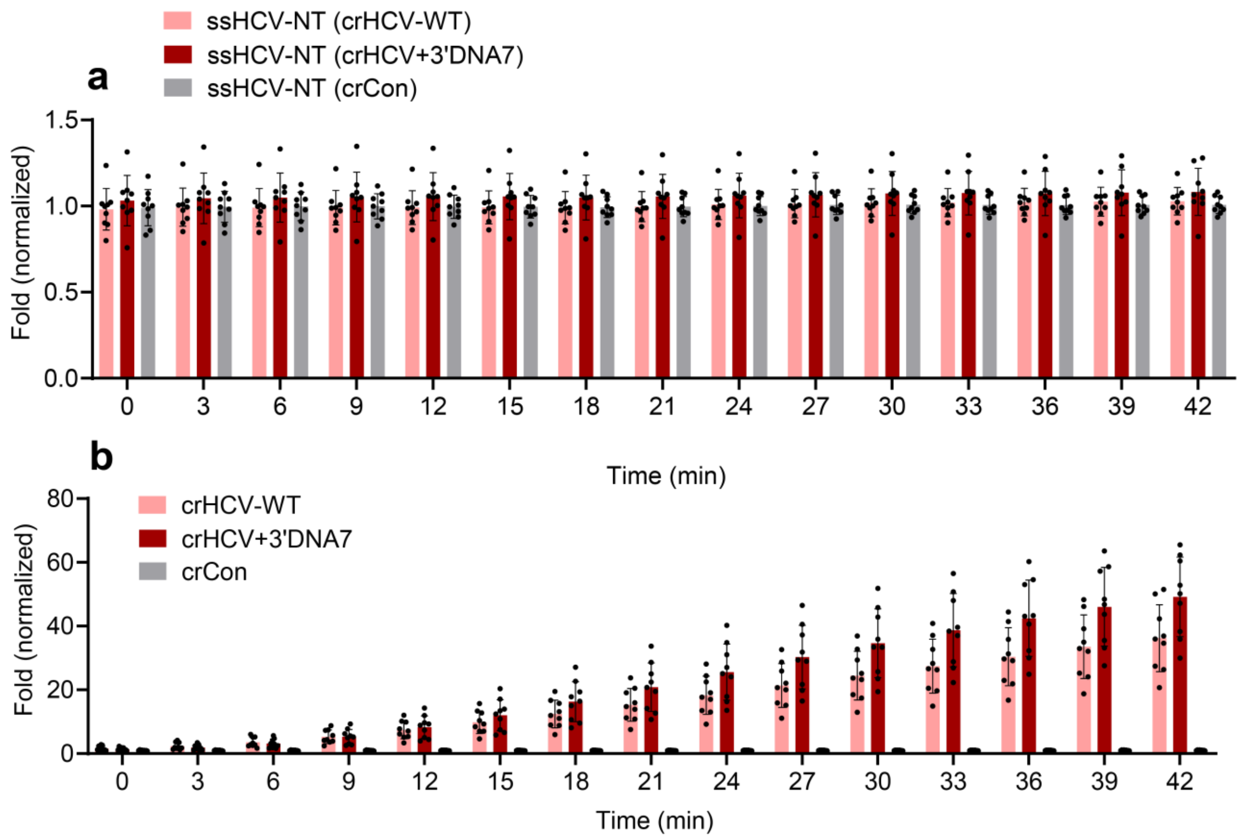

Supplementary Figure 24. Trans-cleavage activity of LbCas12a over time in the presence of 10 nM (100 pmols) of HCV non-target ssDNA (**a**) and HCV dsDNA (**b**). Using engineered crRNA with optimized CRISPR assay, detection of HCV target ssDNA was found to be 29 amols (290 fM, 100 uL) at 30 min, without target amplification. Error bars represent mean ± SEM, where n = 9 (three technical replicates examined over three independent experiments).

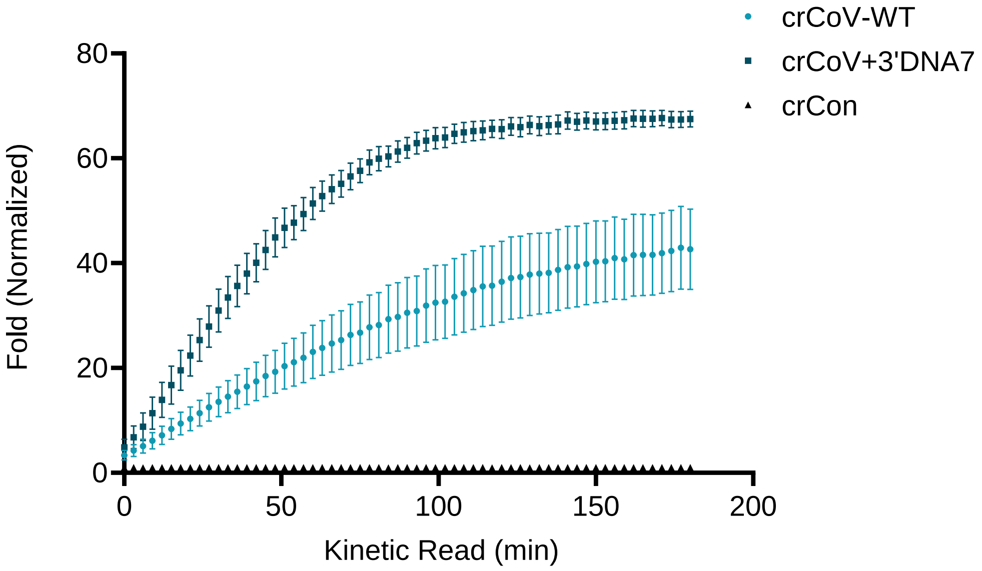

Supplementary Figure 25. Trans-cleavage activity of LbCas12a over time in the presence of 10 nM (100 pmols) of SARS-CoV-2 dsDNA target (bottom). Using engineered crRNA, containing 3’DNA7, and optimized CRISPR assay, the detection of SARS-COV-2 target ssDNA was found to be significantly faster compared to the wild type crRNA, without target amplification. Error bars represents mean ± SEM, where n = 6 (three technical replicates examined over two independent experiments).

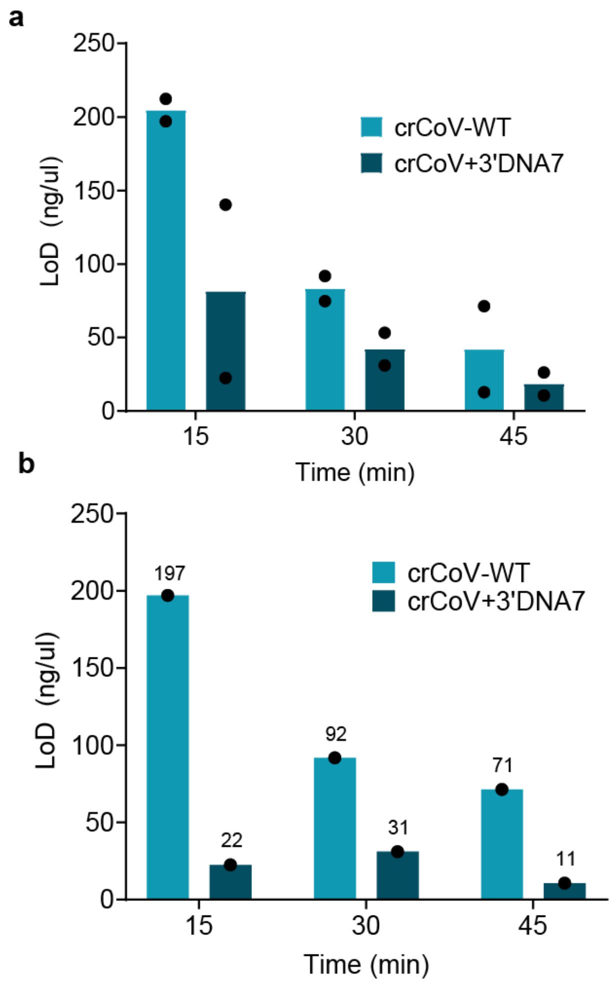

Supplementary Figure 26. Determination of limit of detection for SARS-CoV-2 RNA using LbCas12a. To detect RNA, first a reverse transcriptase step was formed to convert RNA into DNA/RNA heteroduplex. The heteroduplex was detected by using an optimized CRISPR assay over time using either wild type crRNA (crCoV-WT) or engineered crRNA containing 3'DNA7 modifications (crCoV+3'DNA7). The limit of detection for SARS-CoV-2 target RNA was found to be significantly lower with crCoV+3'DNA7, compared to the wild type crRNA, without target amplification. (a) LoD were plotted from two independent experiments. (**b**) is representative data with the lowest limit of detection from (a).

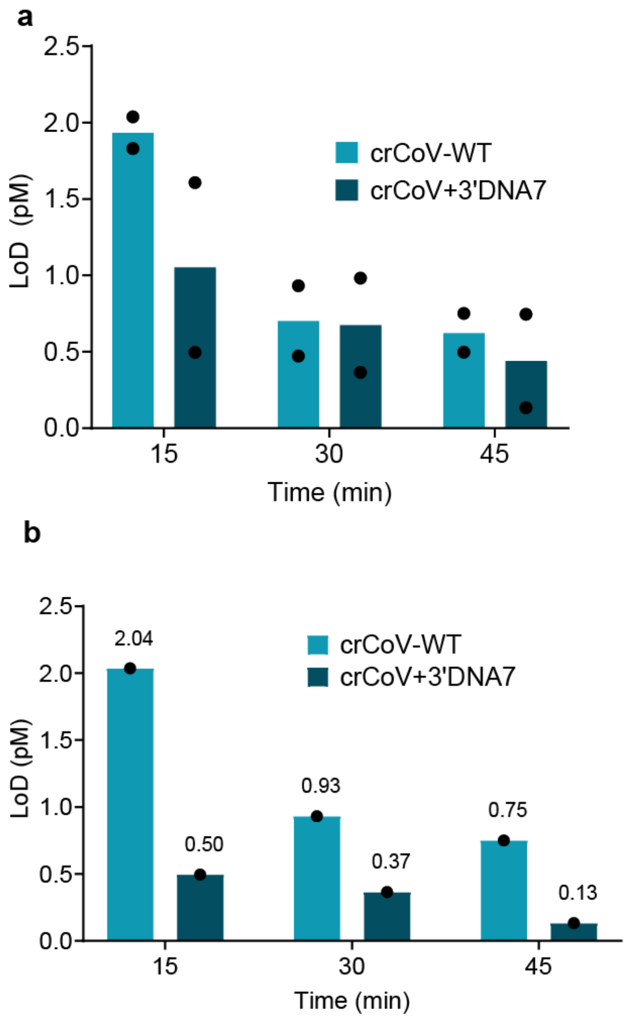

Supplementary Figure 27. Determination of lowest limit of detection for SARS-COV-2 dsDNA using LbCas12a with an optimized CRISPR assay over time using either wild type crRNA (crCoV-WT) or engineered crRNA containing 3'DNA7 modifications (crCoV+3'DNA7). The limit of detection for SARS-COV-2 target RNA was found to be significantly lower with crCoV+3'DNA7 (130 fM), compared to the crCoV-WT (750 fM) within 45 minutes, without target amplification. (a) LoD were plotted from two independent experiments. (**b**) is representative data with the lowest limit of detection from (a).

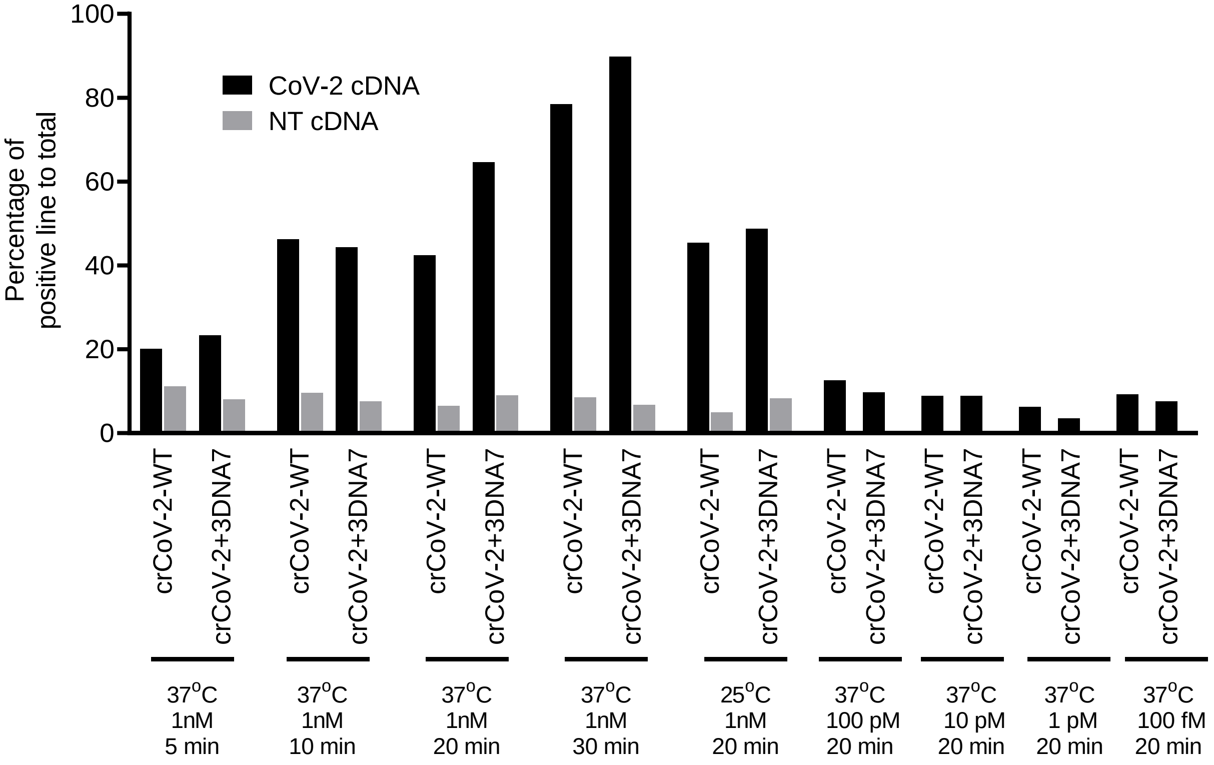

Supplementary Figure 28. Band intensity analysis of paper-strip test of LbCas12a targeting SARS-CoV-2. In this experiment, incubation time and temperature were varied (see materials and methods for more details). The paper-strips were scanned under Typhoon Amersham (GE healthcare) and analyzed using ImageJ.

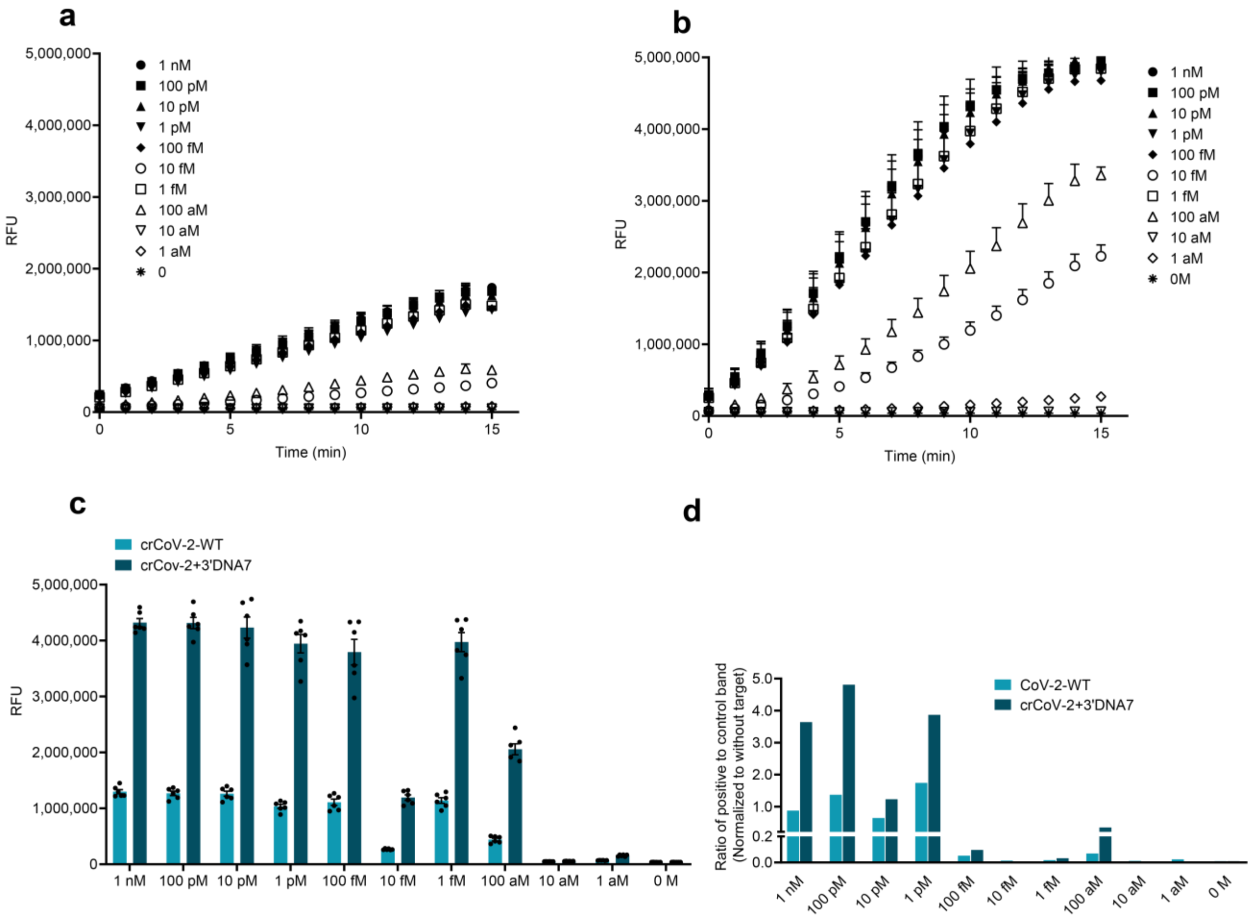

Supplementary Figure 29. Trans-cleavage activity of LbCas12a targeting SARS-CoV-2 with a RT-LAMP preamplification step via fluorescence-quencher reporter assay with FAM-TA**.** The data shown above are raw fluorescence signal measured by the fluorescence microplate reader Biotek Synergy 2. (a) and (b) are kinetics data in 15 min of crCoV-2-WT and crCoV-2+3'DNA7. In this experiment, the concentration of FQ reporter was doubled compared to previous experiments (100 nM). (c) is a single-point fluorescence signal extracted from (a) and (b). (d) Lateral flow assay with RT-LAMP preamplification step. The paper strips were scanned and analyzed using imageJ. Error bars represent in (a), (b), and (c) represent mean ± SEM, where n = 6 (three technical replicates examined over two independent experiments).

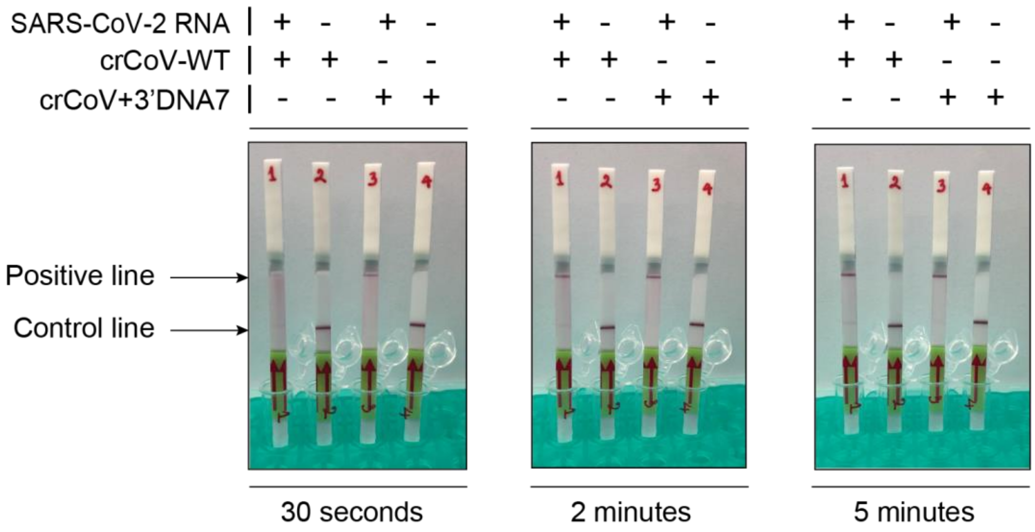

Supplementary Figure 30. A representation of time lapse pictures of the lateral flow assay targeting SARS-CoV-2 RNA N gene. The paper strips were immediately dipped into the LbCas12a reaction after the incubation. The testing concentration was 1 nM SARS-CoV-2 RNA.

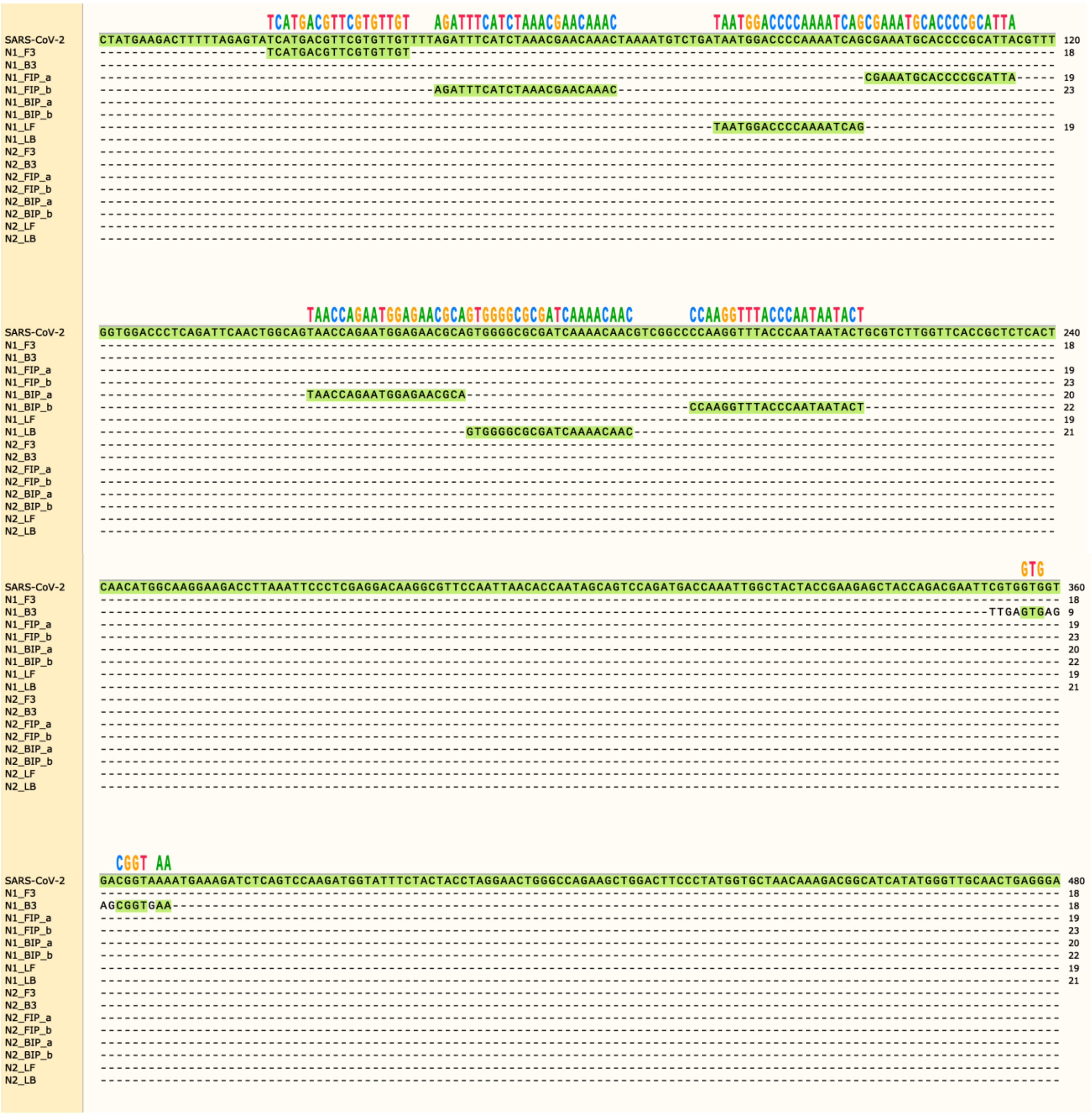
TCATGACGTTCGTGTTGT AGATTTCATCTAAACGAACAAAC TAATGGACCCCAAAATCAGCGAAATGCACCCCGCATTA
SARS-CoV-2 CTATGAAGACTTTTTAGAGTATCATGACGTTCGTGTTGTTTTAGATTTCATCTAAACGAACAAACTAAAATGTCTGATAATGGACCCCAAAATCAGCGAAATGCACCCCGCATTACGTTT 120
N1_F3 TCATGACGTTCGTGTTGT 18
N1_B3
N1_FIP_a CGAAATGCACCCCGCATTA 19
N1_FIP_b AGATTTCATCTAAACGAACAAAC 23
N1_BIP_a
N1_BIP_b
N1_LF TAATGGACCCCAAAATCAG 19
N1_LB
N2_F3
N2_B3
N2_FIP_a
N2_FIP_b
N2_BIP_a
N2_BIP_b
N2_LF
N2_LB
TAACCAGAATGGAGAACGCAGTGGGGCGCGATCAAAACAAC CCAAGGTTTACCCAATAATACT
SARS-CoV-2 GGTGGACCCTCAGATTCAACTGGCAGTAACCAGAATGGAGAACGCAGTGGGGCGCGATCAAAACAACGTCGGCCCCAAGGTTTACCCAATAATACTGCGTCTTGGTTCACCGCTCTCACT 240
N1_F3 18
N1_B3
N1_FIP_a 19
N1_FIP_b 23
N1_BIP_a TAACCAGAATGGAGAACGCA 20
N1_BIP_b CCAAGGTTTACCCAATAATACT 22
N1_LF 19
N1_LB GTGGGGCGCGATCAAAACAAC 21
N2_F3
N2_B3
N2_FIP_a
N2_FIP_b
N2_BIP_a
N2_BIP_b
N2_LF
N2_LB
GTG
SARS-CoV-2 CAACATGGCAAGGAAGACCTTAAATTCCCTCGAGGACAAGGCGTTCCAATTAACACCAATAGCAGTCCAGATGACCAAATTGGCTACTACCGAAGAGCTACCAGACGAATTCGTGGTGGT 360
N1_F3 18
N1_B3 TTGAGTGAG 9
N1_FIP_a 19
N1_FIP_b 23
N1_BIP_a 20
N1_BIP_b 22
N1_LF 19
N1_LB 21
N2_F3
N2_B3
N2_FIP_a
N2_FIP_b
N2_BIP_a
N2_BIP_b
N2_LF
N2_LB
CGGT AA
SARS-CoV-2 GACGGTAAAATGAAAGATCTCAGTCCAAGATGGTATTTCTACTACCTAGGAACTGGGCCAGAAGCTGGACTTCCCTATGGTGCTAACAAAGACGGCATCATATGGGTTGCAACTGAGGGA 480
N1_F3 18
N1_B3 AGCGGTGAA 18
N1_FIP_a 19
N1_FIP_b 23
N1_BIP_a 20
N1_BIP_b 22
N1_LF 19
N1_LB 21
N2_F3
N2_B3
N2_FIP_a
N2_FIP_b
N2_BIP_a
N2_BIP_b
N2_LF
N2_LB

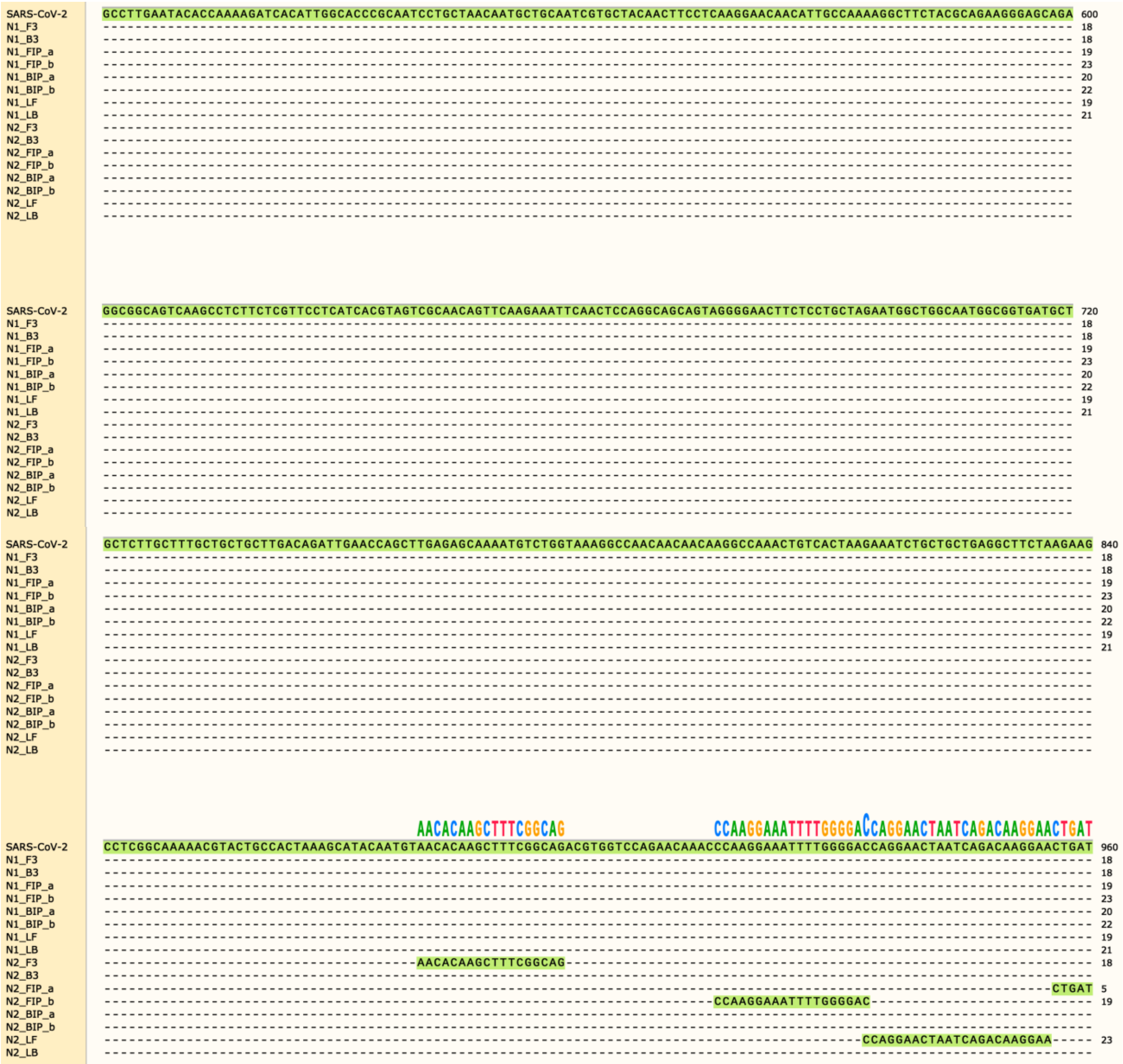
SARS-CoV-2
N1_F3
N1_B3
N1_FIP_a
N1_FIP_b
N1_BIP_a
N1_BIP_b
N1_LF
N1_LB
N2_F3
N2_B3
N2_FIP_a
N2_FIP_b
N2_BIP_a
N2_BIP_b
N2_LF
N2_LB
GCCTTGAATACACCAAAAGATCACATTGGCACCCGCAATCCTGCTAACAATGCTGCAATCGTGCTACAACTTCCTCAAGGAACAACATTGCCAAAAGGCTTCTACGCAGAAGGGAGCAGA 600
GGCGGCAGTCAAGCCTCTTCTCGTTCCTCATCACGTAGTCGCAACAGTTCAAGAAATTCAACTCCAGGCAGCAGTAGGGGAACTTCTCCTGCTAGAATGGCTGGCAATGGCGGTGATGCT 720
GCTCTTGCTTTGCTGCTGCTTGACAGATTGAACCAGCTTGAGAGCAAAATGTCTGGTAAAGGCCAACAACAACAAGGCCAAACTGTCACTAAGAAATCTGCTGCTGAGGCTTCTAAGAAG 840
AACACAAGCTTTCGGCAG
CCAAGGAAATTTTGGGGACCAGGAACTAATCAGACAAGGAACTGAT
CCTCGGCAAAAACGTACTGCCACTAAAGCATACAATGTAACACAAGCTTTCGGCAGACGTGGTCCAGAACAAACCCAAGGAAATTTTGGGGACCAGGAACTAATCAGACAAGGAACTGAT 960
18
18
19
23
20
22
19
21
AACACAAGCTTTCGGCAG 18
CTGAT 5
CCAAGGAAATTTTGGGGAC 19
CCAGGAACTAATCAGACAAGGAA 23

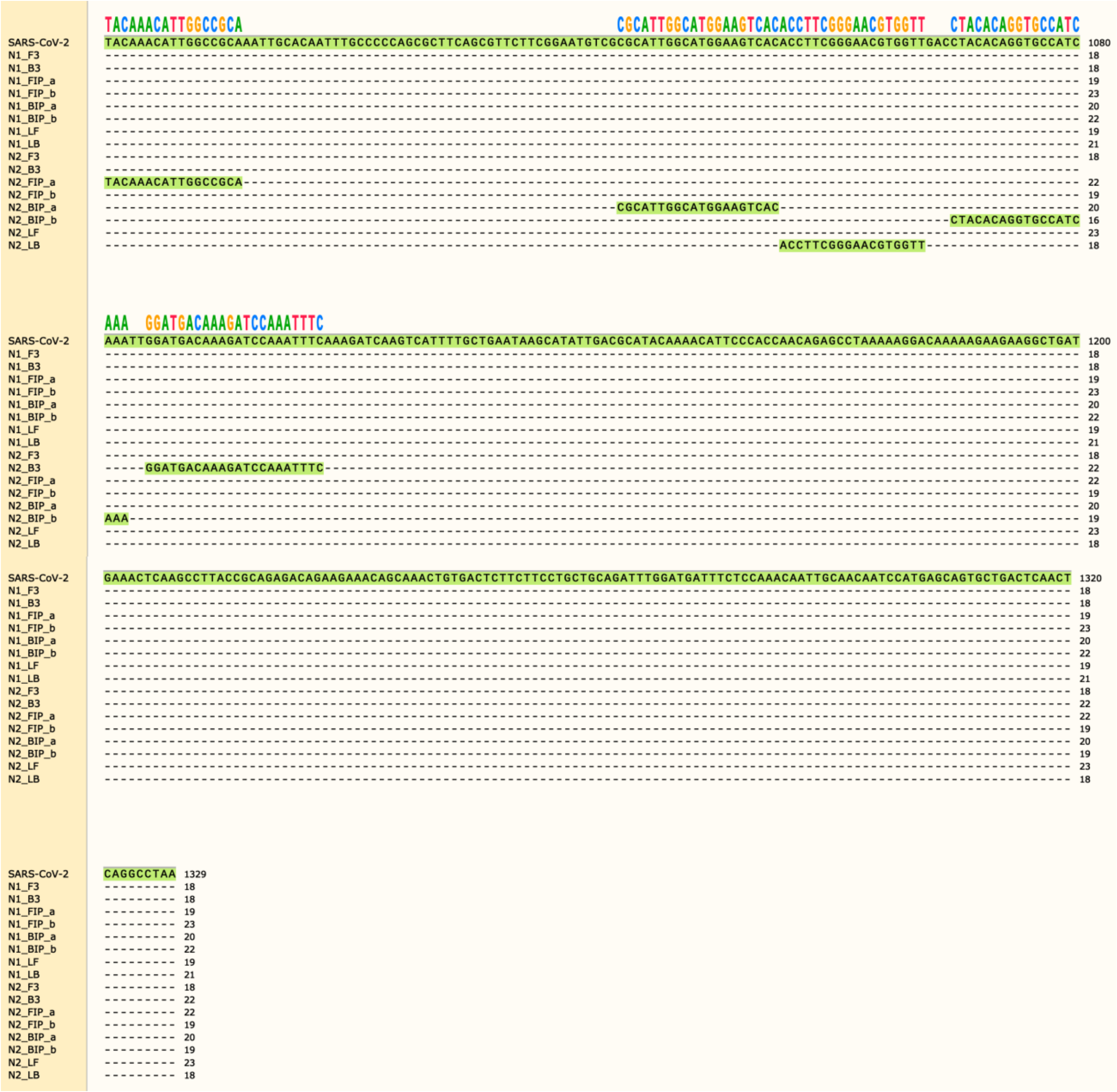

Supplementary Figure 31. Sequence alignment of two sets of LAMP primers against SARS-CoV-2 N gene. Each of two LAMP primer sets N1 and N2 targets 8 different regions within the N gene. F3, B3, FIP, BIP, LF, and LB are primers. FIP is comprised of FIP_a and FIP_b. BIP is comprised of BIP_a and BIP_b. Alignment was performed using TCOFFEE[1], viewed and exported via Snapgene.

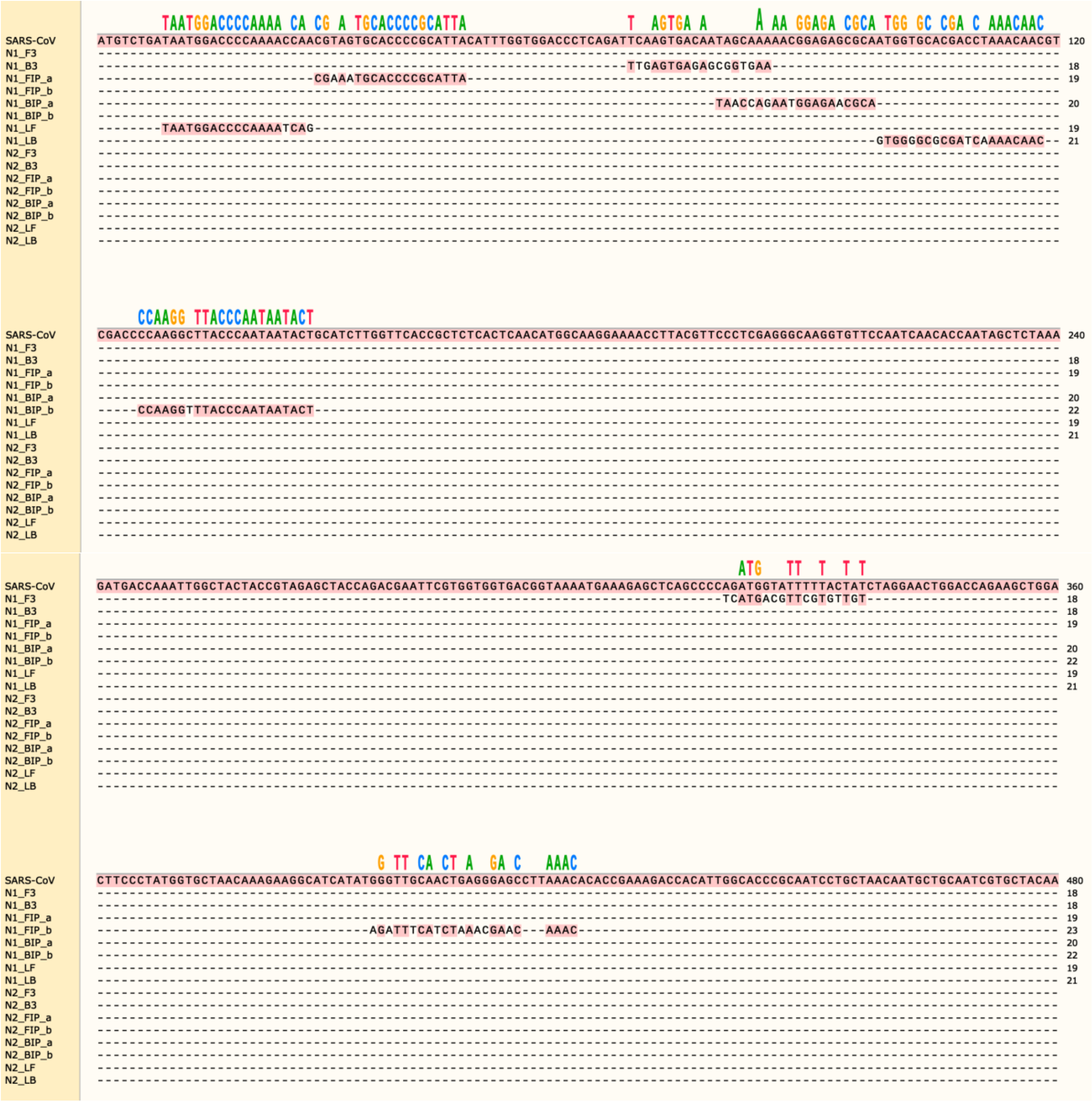
SARS-CoV
N1_F3
N1_B3
N1_FIP_a
N1_FIP_b
N1_BIP_a
N1_BIP_b
N1_LF
N1_LB
N2_F3
N2_B3
N2_FIP_a
N2_FIP_b
N2_BIP_a
N2_BIP_b
N2_LF
N2_LB
120
240
360
480

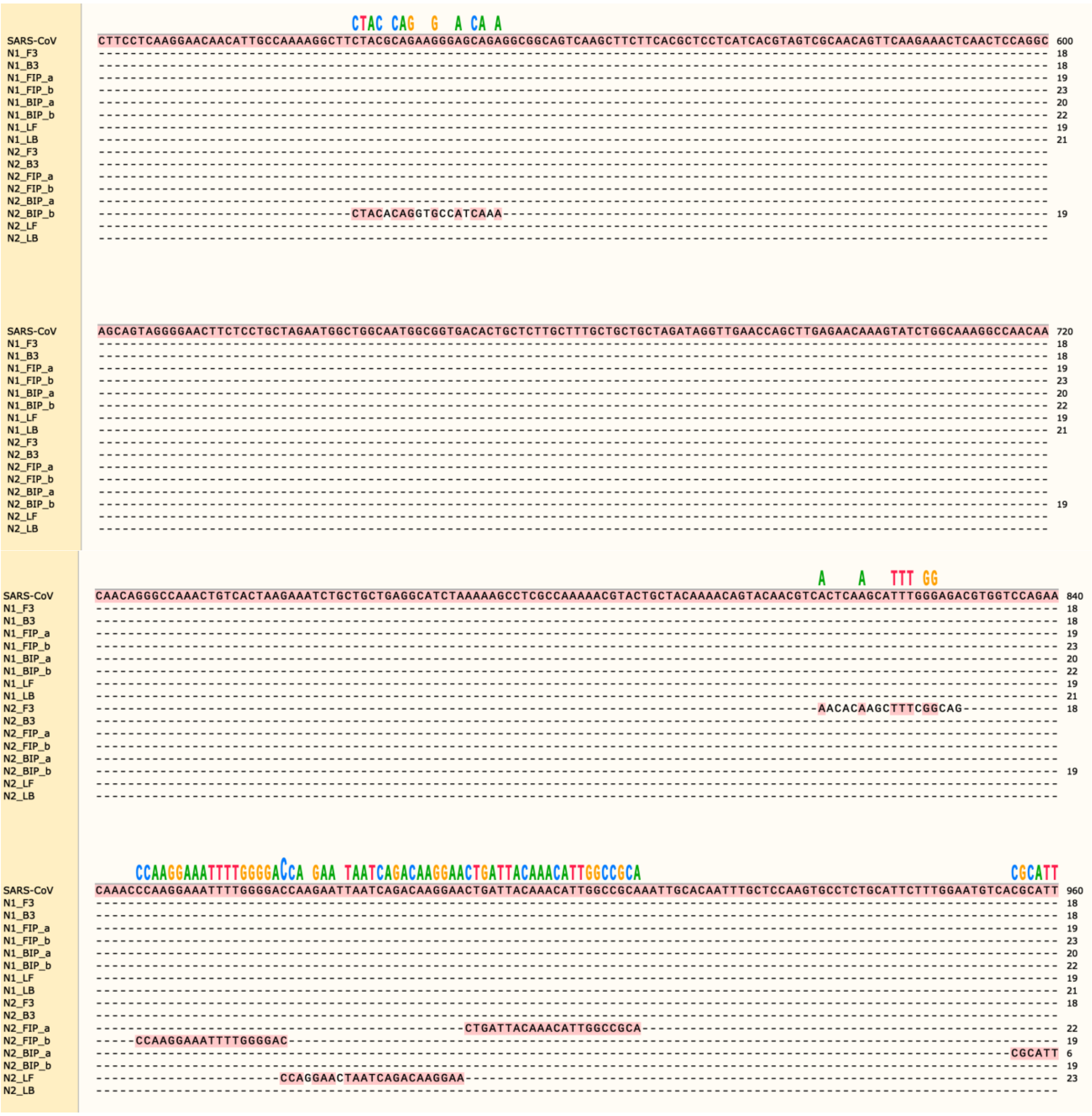
SARS-CoV
N1_F3
N1_B3
N1_FIP_a
N1_FIP_b
N1_BIP_a
N1_BIP_b
N1_LF
N1_LB
N2_F3
N2_B3
N2_FIP_a
N2_FIP_b
N2_BIP_a
N2_BIP_b
N2_LF
N2_LB
CTACACAGGTGCCATCAAA
AACACAAGCTTTCGGCAG
CTGATTACAAACATTGGCCGCA
CCAAGGAAATTTTGGGGAC
CGCATT
CCAGGAACTAATCAGACAAGGAA
600
720
840
960

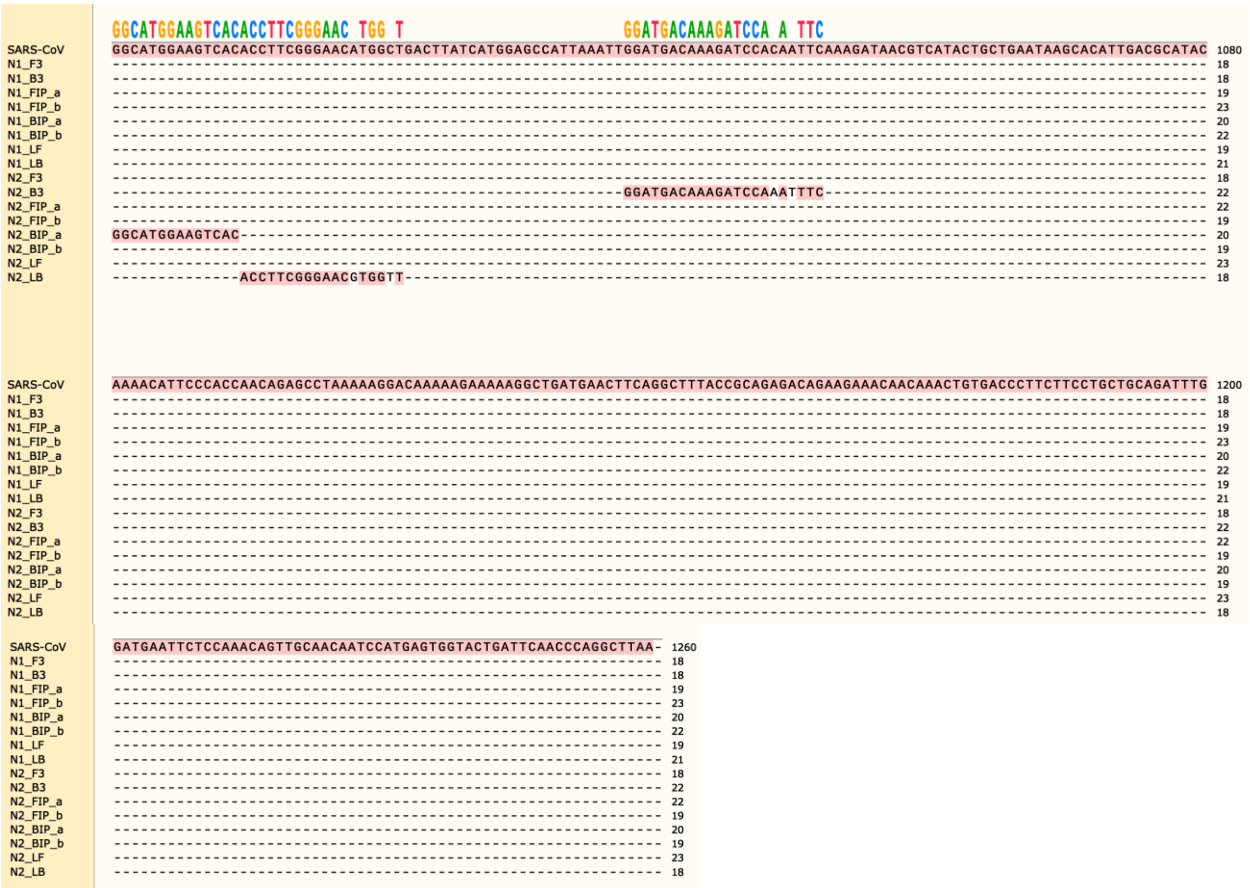

Supplementary Figure 32. Sequence alignment of two sets of LAMP primers against highly similar pathogen SARS-CoV N gene. Each of two LAMP primer sets N1 and N2 targets 8 different regions within the N gene. Sequence mismatches were found for multiple primers of the N1 and N2 sets. F3, B3, FIP, BIP, LF, and LB are primers. FIP is comprised of FIP_a and FIP_b. BIP is comprised of BIP_a and BIP_b. Alignment was performed using TCOFFEE[1], viewed and exported via Snapgene.

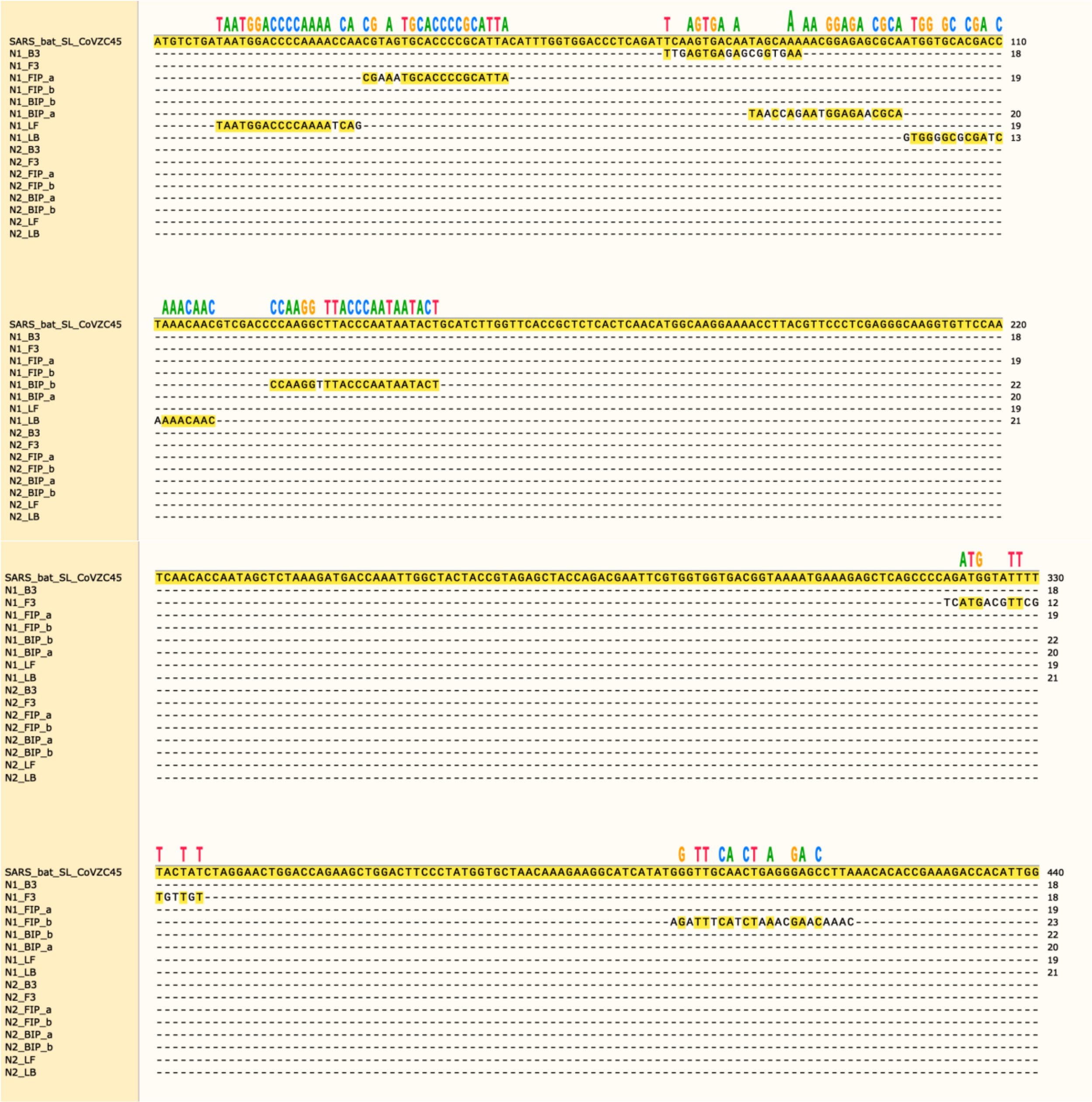

SARS_bat_SL_CoVZC45
N1_B3
N1_F3
N1_FIP_a
N1_FIP_b
N1_BIP_b
N1_BIP_a
N1_LF
N1_LB
N2_B3
N2_F3
N2_FIP_a
N2_FIP_b
N2_BIP_a
N2_BIP_b
N2_LF
N2_LB
ATGTCTGATAATGGACCCCAAAACCAACGTAGTGCACCCCGCATTACATTTGGTGGACCCTCAGATTCAAGTGACAATAGCAAAAACGGAGAGCGCAATGGTGCACGACC 110
TTGAGTGAGAGCGGTGAA 18
CGAAATGCACCCCGCATTA 19
TAACCAGAATGGAGAACGCA 20
TAATGGACCCCAAAATCAG 19
GTGGGGCGCGATC 13
TAAACAACGTCGACCCCAAGGCTTACCCAATAATACTGCATCTTGGTTCACCGCTCTCACTCAACATGGCAAGGAAAACCTTACGTTCCCTCGAGGGCAAGGTGTTCCAA 220
CCAAGGTTTACCCAATAATACT 22
AAAACAAC 21
TCAACACCAATAGCTCTAAAGATGACCAAATTGGCTACTACCGTAGAGCTACCAGACGAATTCGTGGTGGTGACGGTAAAATGAAAGAGCTCAGCCCCAGATGGTATTTT 330
TCATGACGTTCG 12
TACTATCTAGGAACTGGACCAGAAGCTGGACTTCCCTATGGTGCTAACAAAGAAGGCATCATATGGGTTGCAACTGAGGGAGCCTTAAACACACCGAAAGACCACATTGG 440
TGTTGT 18
AGATTTCATCTAAACGAACAAAC 23

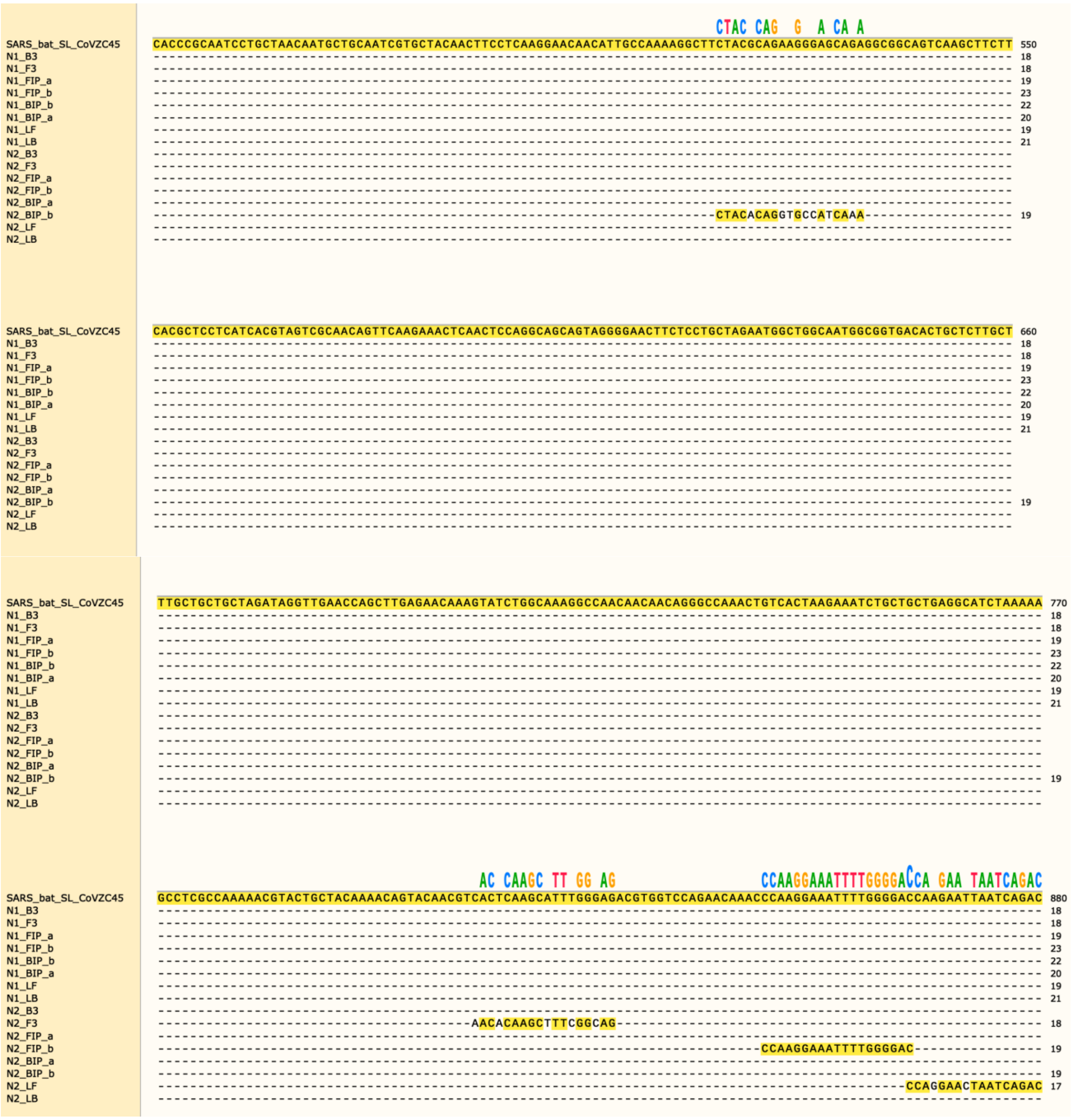

CTAC CAG G A CA A
SARS_bat_SL_CoVZC45 CACCCGCAATCCTGCTAACAATGCTGCAATCGTGCTACAACTTCCTCAAGGAACAACATTGCCAAAAGGCTTCTACGCAGAAGGGAGCAGAGGCGGCAGTCAAGCTTCTT 550
N1_B3 18
N1_F3 18
N1_FIP_a 19
N1_FIP_b 23
N1_BIP_b 22
N1_BIP_a 20
N1_LF 19
N1_LB 21
N2_B3
N2_F3
N2_FIP_a
N2_FIP_b
N2_BIP_a
N2_BIP_b CTACACAGGTGCCATCAA 19
N2_LF
N2_LB
SARS_bat_SL_CoVZC45 CACGCTCCTCATCACGTAGTCGCAACAGTTCAAGAAACTCAACTCCAGGCAGCAGTAGGGGAACTTCTCCTGCTAGAATGGCTGGCAATGGCGGTGACACTGCTCTTGCT 660
N1_B3 18
N1_F3 18
N1_FIP_a 19
N1_FIP_b 23
N1_BIP_b 22
N1_BIP_a 20
N1_LF 19
N1_LB 21
N2_B3
N2_F3
N2_FIP_a
N2_FIP_b
N2_BIP_a
N2_BIP_b 19
N2_LF
N2_LB
SARS_bat_SL_CoVZC45 TTGCTGCTGCTAGATAGGTTGAACCAGCTTGAGAACAAAGTATCTGGCAAAGGCCAACAACAACAGGGCCAAACTGTCACTAAGAAATCTGCTGCTGAGGCATCTAAAAA 770
N1_B3 18
N1_F3 18
N1_FIP_a 19
N1_FIP_b 23
N1_BIP_b 22
N1_BIP_a 20
N1_LF 19
N1_LB 21
N2_B3
N2_F3
N2_FIP_a
N2_FIP_b
N2_BIP_a
N2_BIP_b 19
N2_LF
N2_LB
AC CAAGC TT GG AG
CCAAGGAAATTTTGGGGACCA GAA TAATCAGAC
SARS_bat_SL_CoVZC45 GCCTCGCCAAAAACGTACTGCTACAAAACAGTACAACGTCACTCAAGCATTTGGGAGACGTGGTCCAGAACAAACCCAAGGAAATTTTGGGGACCAAGAATTAATCAGAC 880
N1_B3 18
N1_F3 18
N1_FIP_a 19
N1_FIP_b 23
N1_BIP_b 22
N1_BIP_a 20
N1_LF 19
N1_LB 21
N2_B3
N2_F3 AACACAAGCTTTCGGCAG 18
N2_FIP_a
N2_FIP_b CCAAGGAAATTTTGGGGAC 19
N2_BIP_a
N2_BIP_b 19
N2_LF CCAGGAACTAATCAGAC 17
N2_LB

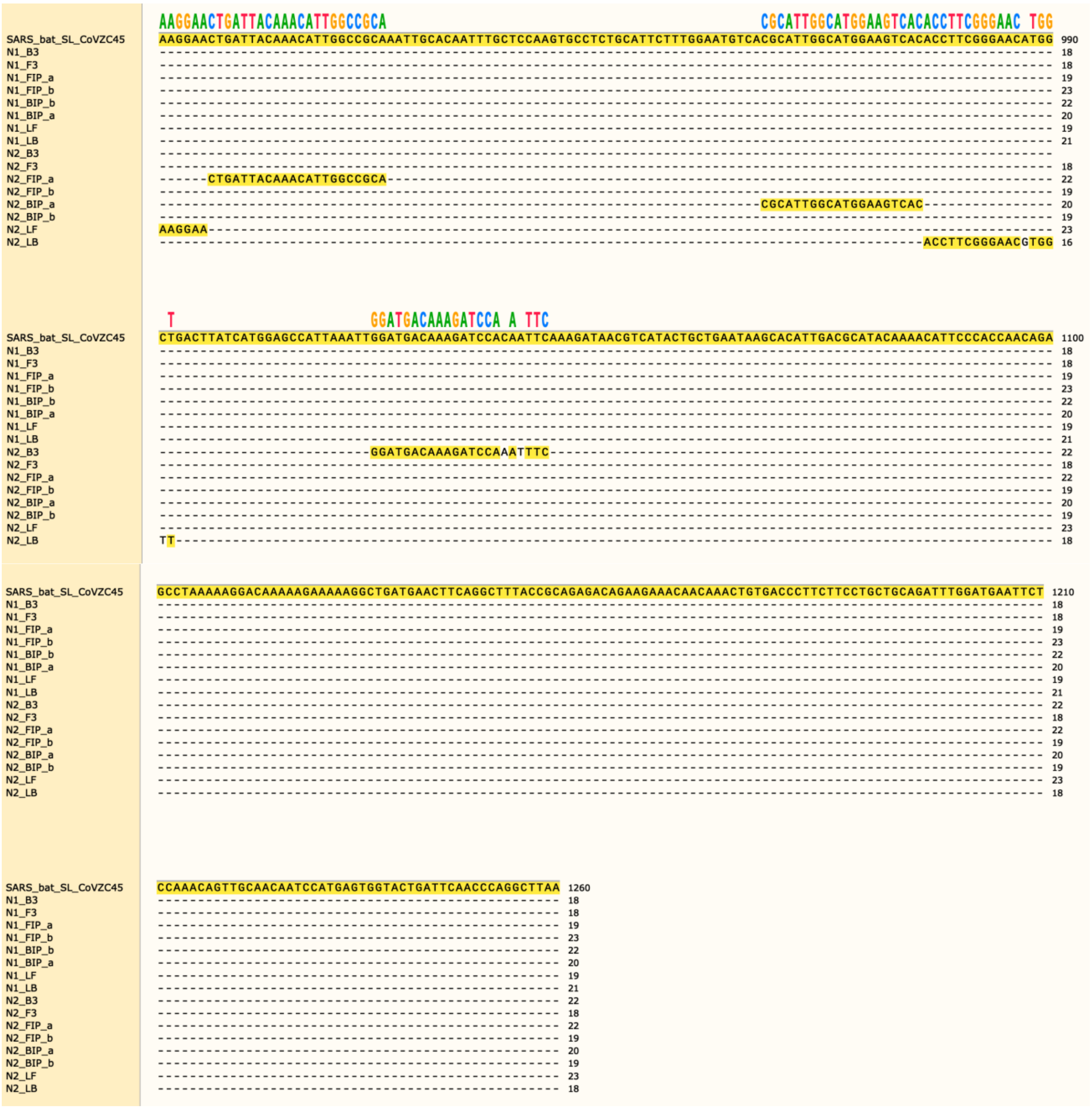

Supplementary Figure 33. Sequence alignment of two sets of LAMP primers against highly similar pathogen SARS-like-bat-SL-CoVZC45 N gene. Each of two LAMP primer sets N1 and N2 targets 8 different regions within the N gene. Sequence mismatches were found for multiple primers of the N1 and N2 sets. F3, B3, FIP, BIP, LF, and LB are primers. FIP is comprised of FIP_a and FIP_b. BIP is comprised of BIP_a and BIP_b. Alignment was performed using TCOFFEE[1], viewed and exported via Snapgene.

Reference

1. Notredame, C., Higgins, D. G. & Heringa, J. T-Coffee: A novel method for fast and accurate multiple sequence alignment. *J Mol Biol* **302**, 205-217, (2000).

CRISPR RNAs (crRNAs)

Selected crRNAs for AsCas12a

Sequence Name	Sequence
AscrCon	/AlTR1/rUrArA rUrUrU rCrUrA rCrUrC rUrUrG rUrArG rArUrC rGrUrU rArArU rCrGrC rGrUrA rUrArA rUrArC rGrG/AlTR2/
AscrGFP1	/AltR1/rUrArA rUrUrU rCrUrA rCrUrC rUrUrG rUrArG rArUrC rGrUrC rGrCrC rGrUrC rCrArG rCrUrC rGrArC rC/AltR2/
AscrGFP2-WT	/AltR1/rUrArA rUrUrU rCrUrA rCrUrC rUrUrG rUrArG rArUrC rUrCrA rGrGrG rCrGrG rArCrU rGrGrG rUrGrC rU/AltR2/
AscrGFP2-WT-no-Alt	rUrArA rUrUrU rCrUrA rCrUrC rUrUrG rUrArG rArUrC rUrCrA rGrGrG rCrGrG rArCrU rGrGrG rUrGrC rU
AscrGFP2+3'DNA7	rUrArA rUrUrU rCrUrA rCrUrC rUrUrG rUrArG rArUrC rUrCrA rGrGrG rCrGrG rArCrU rGrGrG rUrGrC rUTA TTA TT

Selected crRNAs for FnCas12a

FncrGFP2-WT	rUrArA rUrUrU rCrUrA rCrUrG rUrUrG rUrArG rArUrC rUrCrA rGrGrG rCrGrG rArCrU rGrGrG rUrGrC rU
FncrGFP2+3'DNA7	rUrArA rUrUrU rCrUrA rCrUrG rUrUrG rUrArG rArUrC rUrCrA rGrGrG rCrGrG rArCrU rGrGrG rUrGrC rUTA TTA TT
FncrPCA3	rUrArA rUrUrU rCrUrA rCrUrG rUrUrG rUrArG rArUrU rCrArC rCrCrC rUrGrC rCrArU rUrGrA rGrArU rG

Selected crRNAs for LbCas12a

LbcrCon (Neg-Ctrl-LbcrRNA)	rUrArA rUrUrU rCrUrA rCrUrA rArGrU rGrUrA rGrArU rCrGrU rUrArA rUrCrG rCrGrU rArUrA rArUrA rCrGrG
LbcrGFP1	rUrArA rUrUrU rCrUrA rCrUrA rArGrU rGrUrA rGrArU rCrGrU rCrGrC rCrGrU rCrCrA rGrCrU rCrGrA rCrC
LbcrGFP2-WT	rUrArA rUrUrU rCrUrA rCrUrA rArGrU rGrUrA rGrArU rCrUrC rArGrG rGrCrG rGrArC rUrGrG rGrUrG rCrU
LbcrPCA3-WT	rUrArA rUrUrU rCrUrA rCrUrA rArGrU rGrUrA rGrArU rUrCrA rCrCrC rCrUrG rCrCrA rUrUrG rArGrA rUrG
LbcrPCA3+3'DNA7	rUrArA rUrUrU rCrUrA rCrUrA rArGrU rGrUrA rGrArU rUrCrA rCrCrC rCrUrG rCrCrA rUrUrG rArGrA rUrGT ATT ATT
LbcrHIV-WT	rUrArArUrUrUrCrUrArCrUrArArGrUrGrUrArGrArUrCrCrUrUrGrGrUrGrG rGrUrGrCrUrArCrUrCrCrU
LbcrHIV+3'DNA7	rUrArArUrUrUrCrUrArCrUrArArGrUrGrUrArGrArUrCrCrUrUrGrGrUrGrG rGrUrGrCrUrArCrUrCrCrUTATTATT
LbcrHCV-WT	rUrArArUrUrUrCrUrArCrUrArArGrUrGrUrArGrArUrUrGrCrUrCrArUrGrA rUrGrCrArCrGrGrUrCrUrA
LbcrHCV+3'DNA7	rUrArArUrUrUrCrUrArCrUrArArGrUrGrUrArGrArUrUrGrCrUrCrArUrGr ArUrGrCrArCrGrGrUrCrUrATATTATT
N1:crCoV-2-WT	rUrArArUrUrUrCrUrArCrUrArArGrUrGrUrArGrArUrGrUrGrGrArCrCrCr UrCrArGrArUrUrCrArArCrU
N1:crCoV-2+3'DNA7	rUrArArUrUrUrCrUrArCrUrArArGrUrGrUrArGrArUrGrUrGrGrArCrCrCr UrCrArGrArUrUrCrArArCrUTATTATT
N2:crCoV-2-WT	rUrArArUrUrUrCrUrArCrUrArArGrUrGrUrArGrArUrCrCrCrCrCrArGrCrGr CrUrUrCrArGrCrGrUrUrC

N2:crCoV-2+3'DNA7	rUrArArUrUrUrCrUrArCrUrArArGrUrGrUrArGrArUrCrCrCrCrArGrCrGrCrUrUrCrArGrCrGrUrUrCTATTATT

3' DNA modified crGFP2 for LbCas12a

**Sequence Name**	**Sequence**
LbcrGFP2+3'DNA7	rUrArA rUrUrU rCrUrA rCrUrA rArGrU rGrUrA rGrArU rCrUrC rArGrG rGrCrG rGrArC rUrGrG rGrUrG rCrUT ATT ATT
LbcrGFP2+3'DNA13	rUrArA rUrUrU rCrUrA rCrUrA rArGrU rGrUrA rGrArU rCrUrC rArGrG rGrCrG rGrArC rUrGrG rGrUrG rCrUT ATT ATT ATT ATT
LbcrGFP2+3'DNA19	rUrArA rUrUrU rCrUrA rCrUrA rArGrU rGrUrA rGrArU rCrUrC rArGrG rGrCrG rGrArC rUrGrG rGrUrG rCrUT ATT ATT ATT ATT ATT ATT
LbcrGFP2+3'DNA31	rUrArA rUrUrU rCrUrA rCrUrA rArGrU rGrUrA rGrArU rCrUrC rArGrG rGrCrG rGrArC rUrGrG rGrUrG rCrUT ATT ATT ATT ATT ATT ATT ATT ATT ATT ATT
LbcrGFP2+3'DNA7(GC)	rUrArA rUrUrU rCrUrA rCrUrA rArGrU rGrUrA rGrArU rCrUrC rArGrG rGrCrG rGrArC rUrGrG rGrUrG rCrUC GCC GCC

5' DNA modified crGFP2 for LbCas12a

**Sequence Name**	**Sequence**
LbcrGFP2+5'DNA7	TTA TTA TrUrA rArUrU rUrCrU rArCrU rArArG rUrGrU rArGrA rUrCrU rCrArG rGrGrC rGrGrA rCrUrG rGrGrU rGrCrU
Tru-LbcrGFP2+5'DNA7	TTA TTA TrArA rUrUrU rCrUrA rCrUrA rArGrU rGrUrA rGrArU rCrUrC rArGrG rGrCrG rGrArC rUrGrG rGrUrG rCrU
LbcrGFP2+5'DNA13	TTA TTA TTA TTA TrUrA rArUrU rUrCrU rArCrU rArArG rUrGrU rArGrA rUrCrU rCrArG rGrGrC rGrGrA rCrUrG rGrGrU rGrCrU
LbcrGFP2+5'DNA19	TTA TTA TTA TTA TTA TTA TrUrA rArUrU rUrCrU rArCrU rArArG rUrGrU rArGrA rUrCrU rCrArG rGrGrC rGrGrA rCrUrG rGrGrU rGrCrU
LbcrGFP2+5'DNA31	TTA TTA TTA TTA TTA TTA TTA TTA TTA TTA TrUrA rArUrU rUrCrU rArCrU rArArG rUrGrU rArGrA rUrCrU rCrArG rGrGrC rGrGrA rCrUrG rGrGrU rGrCrU

3' PSDNA modified crGFP2 for LbCas12a

**Sequence Name**	**Sequence**
LbcrGFP2+3'PSDNA7	rUrArA rUrUrU rCrUrA rCrUrA rArGrU rGrUrA rGrArU rCrUrC rArGrG rGrCrG rGrArC rUrGrG rGrUrG rCrU*T* A*T*T* A*T*T
LbcrGFP2+3'PSDNA13	rUrArA rUrUrU rCrUrA rCrUrA rArGrU rGrUrA rGrArU rCrUrC rArGrG rGrCrG rGrArC rUrGrG rGrUrG rCrU*T* A*T*T* A*T*T* A*T*T* A*T*T
LbcrGFP2+3'PSDNA19	rUrArA rUrUrU rCrUrA rCrUrA rArGrU rGrUrA rGrArU rCrUrC rArGrG rGrCrG rGrArC rUrGrG rGrUrG rCrU*T* A*T*T* A*T*T* A*T*T* A*T*T* A*T*T* A*T*T
LbcrGFP+6DNA+1PS-3'	rUrArA rUrUrU rCrUrA rCrUrA rArGrU rGrUrA rGrArU rCrUrC rArGrG rGrCrG rGrArC rUrGrG rGrUrG rCrUT ATT AT*T
LbCrGFP+5DNA+2PS-3'	rUrArA rUrUrU rCrUrA rCrUrA rArGrU rGrUrA rGrArU rCrUrC rArGrG rGrCrG rGrArC rUrGrG rGrUrG rCrUT ATT A*T*T

LbCrGFP+4DNA+3PS-3'	rUrArA rUrUrU rCrUrA rCrUrA rArGrU rGrUrA rGrArU rCrUrC rArGrG rGrCrG rGrArC rUrGrG rGrUrG rCrUT ATT* A*T*T
LbCrGFP+3DNA+4PS-3'	rUrArA rUrUrU rCrUrA rCrUrA rArGrU rGrUrA rGrArU rCrUrC rArGrG rGrCrG rGrArC rUrGrG rGrUrG rCrUT AT*T* A*T*T
LbCrGFP+2DNA+5PS-3'	rUrArA rUrUrU rCrUrA rCrUrA rArGrU rGrUrA rGrArU rCrUrC rArGrG rGrCrG rGrArC rUrGrG rGrUrG rCrUT A*T*T* A*T*T
LbCrGFP+1DNA+6PS-3'	rUrArA rUrUrU rCrUrA rCrUrA rArGrU rGrUrA rGrArU rCrUrC rArGrG rGrCrG rGrArC rUrGrG rGrUrG rCrUT* A*T*T* A*T*T

5’ PSDNA modified crGFP2 for LbCas12a

Sequence Name	Sequence
LbcrGFP2+5'PSDNA7	T*T*A* T*T*A* T*rUrA rArUrU rUrCrU rArCrU rArArG rUrGrU rArGrA rUrCrU rCrArG rGrGrC rGrGrA rCrUrG rGrGrU rGrCrU
LbcrGFP2+5'PSDNA13	T*T*A* T*T*A* T*T*A* T*T*A* T*rUrA rArUrU rUrCrU rArCrU rArArG rUrGrU rArGrA rUrCrU rCrArG rGrGrC rGrGrA rCrUrG rGrGrU rGrCrU
LbcrGFP2+5'PSDNA19	T*T*A* T*T*A* T*T*A* T*T*A* T*T*A* T*T*A* T*rUrA rArUrU rUrCrU rArCrU rArArG rUrGrU rArGrA rUrCrU rCrArG rGrGrC rGrGrA rCrUrG rGrGrU rGrCrU

3’ RNA modified crGFP2 for LbCas12a

Sequence Name	Sequence
LbcrGFP2+3'RNA7	rUrArA rUrUrU rCrUrA rCrUrA rArGrU rGrUrA rGrArU rCrUrC rArGrG rGrCrG rGrArC rUrGrG rGrUrG rCrUrU rArUrU rArUrU
LbcrGFP2+3'RNA13	rUrArA rUrUrU rCrUrA rCrUrA rArGrU rGrUrA rGrArU rCrUrC rArGrG rGrCrG rGrArC rUrGrG rGrUrG rCrUrU rArUrU rArUrU rArUrU rArUrU
LbcrGFP2+3'RNA19	rUrArA rUrUrU rCrUrA rCrUrA rArGrU rGrUrA rGrArU rCrUrC rArGrG rGrCrG rGrArC rUrGrG rGrUrG rCrUrU rArUrU rArUrU rArUrU rArUrU rArUrU rArUrU

5’ RNA modified crGFP2 for LbCas12a

Sequence Name	Sequence
LbcrGFP2+5'RNA7	rUrUrA rUrUrA rUrUrA rArUrU rUrCrU rArCrU rArArG rUrGrU rArGrA rUrCrU rCrArG rGrGrC rGrGrA rCrUrG rGrGrU rGrCrU
LbcrGFP2+5'RNA13	rUrUrA rUrUrA rUrUrA rUrUrA rUrUrA rArUrU rUrCrU rArCrU rArArG rUrGrU rArGrA rUrCrU rCrArG rGrGrC rGrGrA rCrUrG rGrGrU rGrCrU
LbcrGFP2+5'RNA19	rUrUrA rUrUrA rUrUrA rUrUrA rUrUrA rUrUrA rUrUrA rArUrU rUrCrU rArCrU rArArG rUrGrU rArGrA rUrCrU rCrArG rGrGrC rGrGrA rCrUrG rGrGrU rGrCrU

Activator DNA&RNA

Sequence Name	Sequence
DD3/PCA3-40nt-T-ssDNA	AGA CTA CAG ACA TCT CAA TGG CAG GGG TGA GAA ATA AGA A
DD3/PCA3-40nt-T-ssDNA – NT	TTC TTA TTT CTC ACC CCT GCC ATT GAG ATG TCT GTA GTC T
TTATT sequence-13mer	TATTATTATTATT
DD3-PCA3-gene-250bp-transcript variant 1	CAA GAT AAA TAA GTG AAG AGC TAG TCC GCT GTG AGT CTC CTC AGT GAC ACA GGG CTG GAT CAC CAT CGA CGG CAC TTT CTG AGT ACT CAG TGC AGC AAA GAA AGA CTA CAG ACA TCT CAA TGG CAG GGG TGA GAA ATA AGA AAG GCT GCT GAC TTT ACC ATC TGA GGC CAC ACA TCT GCT GAA ATG GAG ATA ATT AAC ATC ACT AGA AAC AGC AAG ATG ACA ATA TAA TGT CTA AGT AGT GAC ATG TTT T
LbCas12a-Activator-HIV1-RNA	rUrUrCrUrCrUrCrUrGrCrArCrCrArCrUrCrUrUrCrUrCrUrUrUrGrCrCrUrUrGrGrUrGrGrGrUrGrCrUrArCrUrCrCrUrArArUrGrGrUrUrCrArArUrUrUrU
LbCas12a-HIV-DNA-T-Activator	AAAATTGAACCATTAGGAGTAGCACCCACCAAGGCAAAGAGAAGAGTGGTGCAGAGAGAA
LbCas12a-HIV-DNA-NT-Activator	TTCTCTCTGCACCACTCTTCTCTTTGCCTTGGTGGGTGCTACTCCTAATGGTTCAATTTT
HCV_RNA_reference_Genome (Polyprotein precursor)	rGrCrCrUrUrGrUrGrGrUrArCrUrGrCrCrUrGrArUrArGrGrGrUrGrCrUrUrGrCrGrArGrUrGrCrCrCrCrGrGrGrArGrGrUrCrUrCrGrUrArGrArCrCrGrUrGrCrArUrCrArUrGrArGrCrArCrArArArUrCrCrUrArArArCrCrUrC
HCV_DNA_act_T	CCTCTAATACGACTCACTATAGGCGTTGGGTTGCGAACGGCCTTGTGGTACTGCCTGATAGGGTGCTTGCGAGTGCCCCGGGAGGTCTCGTAGACCGTGCATCATGAGCACAAATCCTAAACCTC
HCV_DNA_act_NT	GAGGTTTAGGATTTGTGCTCATGATGCACGGTCTACGAGACCTCCCGGGGCACTCGCAAGCACCCTATCAGGCAGTACCACAAGGCCGTTCGCAACCCAACGCCTATAGTGAGTCGTATTAGAGG
2019-nCoV_N_Positive Control	Plasmid CAT_10006625_2019-nCoV_N_Positive Control from IDT
MERS-CoV Control	Plasmid MERS-CoV Control from IDT
Bat-SL-CoVZC45 Control	Plasmid SARS-CoV Control from IDT

Activator Primers

Sequence Name	Sequence
GFP-Act-NT-MedC	GGG GTC TTT G/iMe-dC/T /iMe-dC/AG GG/iMe-dC/ GGA /iMe-dC/TG GGT G/iMe-dC/T CAG GTA GTG G
GFP-Act-T-MedC	CCA CTA CCT GAG /iMe-dC/A/iMe-dC/ /iMe-dC//iMe-dC/A GT/iMe-dC/ /iMe-dC/G/iMe-dC/ /iMe-dC//iMe-dC/T GAG CAA AGA CCC C
GFP-40nt-T-heteroDNA-RNA	CCA CTA CCT GrArG rCrArC rCrCrA rGrUrC rCrGrC rCrCrU rGrArG CAA AGA CCC C
GFP-40nt-NT-heteroDNA-RNA	GGG GTC TTT GrCrU rCrArG rGrGrC rGrGrA rCrUrG rGrGrU rGrCrU CAG GTA GTG G
HIV_RNA_Primer_RT-Superscript	AAA ATT GAA CCA TTA GGA GTA GC
HIV_RNA_Primer_RT-MMLV	AAA ATT GAA CCA TTA GG
HIV-RNA-RP	TTC TCT CTG CAC CAC TCT TC
HCV_RP_gblock_also_RT1_LN	GAG GTT TAG GAT TTG TGC TCA T
HCV_RP_gblock_also_RT2_LN	GAG GTT TAG GAT TTG TGC TCA TGA
HCV_FP_1	CCT CTA ATA CGA CTC ACT A
HCV_FP_2	CCT CTA ATA CGA CTC A
HCV_PCR_cDNA_FP1	GCG TTG GGT TGC GAA CGG CC
HCV_PCR_cDNA_FP2	GCG TTG GGT TGC GAA CGG
HIV_RT_cDNA_FP	GCC TTG TGG TAC TGC CTG AT
2019-nCoV_N1_FP	GAC CCC AAA ATC AGC GAA AT
2019-nCoV_N1_RP	TCT GGT TAC TGC CAG TTG AAT CTG
2019-nCoV_N1_T7FP	CCT CTA ATA CGA CTC ACT ATA GGA CCC CAA AAT CAG CGA AAT
2019-nCoV_N1_T7RP	CCT CTA ATA CGA CTC ACT ATA GGT CTG GTT ACT GCC AGT TGA ATC TG
2019-nCoV-N3_FP_LN	GGG AGC CTT GAA TAC ACC AAA A
2019-nCoV_N3_RP_LN	TGT AGC ACG ATT GCA GCA TTG
2019-nCoV_N2_FP_LN	TTA CAA ACA TTG GCC GCA AA
2019-nCoV_N2_RP_LN	GCG CGA CAT TCC GAA GAA
SARS-CoV_FP	ATGTCTGATAATGGACCCCAAA
SARS-CoV_RP	TTAAGCCTGGGTTGAATCAG
MERS-CoV_FP	CACCTCGTGCTGTTTCCTTT
MERS-CoV_RP	ATCATTGGACCAGGCTGAAC
F3_LAMP_N1	TCATGACGTTCGTGTTGT
B3_LAMP_N1	TTGAGTGAGAGCGGTGAA
FIP_LAMP_N1	TAATGCGGGGTGCATTTCGAGATTTCATCTAAACGAACAAAC
BIP_ LAMP_N1	TAACCAGAATGGAGAACGCAAGTATTATTGGGTAAACCTTGG
LF_LAMP_N1	CTGATTTTGGGGTCCATTA
LB_LAMP_N1	GTGGGGCGCGATCAAAACAAC
F3_LAMP_N2	GCTGCTGAGGCTTCTAAG
B3_LAMP_N2	GCGTCAATATGCTTATTCAGC
FIP_LAMP_N2	GCGGCCAATGTTTGTAATCAGTAGACGTGGTCCAGAACAA
BIP_ LAMP_N2	TCAGCGTTCTTCGGAATGTCGCTGTGTAGGTCAACCACG
LF_LAMP_N2	CCTTGTCTGATTAGTTCCTGGT

LB_LAMP_N2	TGGCATGGAAGTCACACC

FQ Substrates and Labeled crRNAs

Sequence Name	Sequence
ssDNA-FQ reporter1	/56-FAM/TTA TT/3IABkFQ/
Oligo 2 FAM-Biotin	/56-FAM/TTA TT/3Bio/
5'Cy5-3'RQ-FQ-Reporter	TAT TA/iCy5/T TAT T/3IAbRQSp/
FQreporter-Hex-IowaFQ	/5HEX/TTA TT/3IABkFQ/
ssDNA-FAM-FQ reporter1	/56-FAM/TTA TT/3IABkFQ/
FAM-GC-richFQ-Reporter	/56-FAM/CCG CC/3IABkFQ/
FQreporter-FAM-ssRNA(rN)-IABFQ	/56-FAM/rCrCrG rCrC/3IABkFQ/
FQ-reporter-FAM-ssRNA(UArich)	/56-FAM/rUrUrA rUrU/3IABkFQ/
LbcrGFP2+3'DNA7-FAM	rUrArA rUrUrU rCrUrA rCrUrA rArGrU rGrUrA rGrArU rCrUrC rArGrG rGrCrG rGrArC rUrGrG rGrUrG rCrUT ATT ATT/36-FAM/
5'FAM-LbcrGFP2	/56-FAM/rUrArA rUrUrU rCrUrA rCrUrA rArGrU rGrUrA rGrArU rCrUrC rArGrG rGrCrG rGrArC rUrGrG rGrUrG rCrU
LbcrGFP2-3'FAM	rUrArA rUrUrU rCrUrA rCrUrA rArGrU rGrUrA rGrArU rCrUrC rArGrG rGrCrG rGrArC rUrGrG rGrUrG rCrU/36-FAM/
5'FAM-LbcrGFP2+3'DNA13	/56-FAM/rUrArA rUrUrU rCrUrA rCrUrA rArGrU rGrUrA rGrArU rCrUrC rArGrG rGrCrG rGrArC rUrGrG rGrUrG rCrUT ATT ATT ATT ATT
LbcrGFP2+3'DNA13-FAM	rUrArA rUrUrU rCrUrA rCrUrA rArGrU rGrUrA rGrArU rCrUrC rArGrG rGrCrG rGrArC rUrGrG rGrUrG rCrUT ATT ATT ATT ATT/36-FAM/
5'HEX-DNA19+LbcrGFP2	/5HEX/TTA TTA TTA TTA TTA TTA TrUrA rArUrU rUrCrU rArCrU rArArG rUrGrU rArGrA rUrCrU rCrArG rGrGrC rGrGrA rCrUrG rGrGrU rGrCrU
LbcrGFP2+DNA-5'FQ-Cy5 (crGFP+5'DNA13+Cy5+DNA6+Iowa Black RQ)	/5IAbRQ/TTA TT/iCy5/A TTA TTA TTA TTA TrUrA rArUrU rUrCrU rArCrU rArArG rUrGrU rArGrA rUrCrU rCrArG rGrGrC rGrGrA rCrUrG rGrGrU rGrCrU
LbcrGFP2+DNA-3'FQ-Cy5 (crGFP+3'DNA7+Cy5+DNA6+Iowa Black RQ)	rUrArA rUrUrU rCrUrA rCrUrA rArGrU rGrUrA rGrArU rCrUrC rArGrG rGrCrG rGrArC rUrGrG rGrUrG rCrUT ATT ATT A/iCy5/TT ATT /3IAbRQSp/

Primers

Sequence Name	Sequence
LbcrGFP2-3'DNA7-Primer-15	AAT AAT AAG CAC CCA GTC CGC C
LbcrGFP2-3'DNA7-Primer-14	AAT AAT AAG CAC CCA GTC CGC
LbcrGFP2-3'DNA7-Primer-13	AAT AAT AAG CAC CCA GTC CG
LbcrGFP2-3'DNA7-Primer-12	AAT AAT AAG CAC CCA GTC C
LbcrGFP2-3'DNA7-Primer-11	AAT AAT AAG CAC CCA GTC
LbcrGFP2-3'DNA7-Primer-10	AAT AAT AAG CAC CCA GT
LbcrGFP2-3'DNA7-Primer-9	AAT AAT AAG CAC CCA G
LbcrGFP2-3'DNA7-Primer-8	AAT AAT AAG CAC CCA
LbcrGFP2-3'DNA7-Primer-7	AAT AAT AAG CAC CC
LbcrGFP2-3'DNA7-Primer-6	AAT AAT AAG CAC C
LbcrGFP2-3'DNA7-Primer-5	AAT AAT AAG CAC
LbcrGFP2-3'DNA7-Primer-4	AAT AAT AAG CA
LbcrGFP2-Primer-15	AGC ACC CAG TCC GCC
LbcrGFP2-Primer-14	AGC ACC CAG TCC GC
LbcrGFP2-Primer-13	AGC ACC CAG TCC G
LbcrGFP2-Primer-12	AGC ACC CAG TCC
LbcrGFP2-Primer-11	AGC ACC CAG TC
LbcrGFP2-Primer-10	AGC ACC CAG T
LbcrGFP2-Primer-9	AGC ACC CAG
LbcrGFP2-Primer-8	AGC ACC CA
RPA-PCA3-FP1	AGT ACT CAG TGC AGC AAA GAA AGA CTA CAG
RPA-PCA3-RP1	ACA TTA TAT TGT CAT CTT GCT GTT TCT AGT GAT
RPA-PCA3-FP2	AGT GAA GAG CTA GTC CGC TGT GAG TCT CCT
RPA-PCA3-RP2	CTG TTT CTA GTG ATG TTA ATT ATC TCC ATT TC
RPA-PCA3-FP3	AAG AGC TAG TCC GCT GTG AGT CTC CTC AGT
RPA-PCA3-RP3	GTT TCT AGT GAT GTT AAT TAT CTC CAT TTC AG
T7-Foward-primer1-RNA	CCT CTA ATA CGA CTC ACT ATA GGA ACG GCA TCA AGG TGA ACT
T7-Foward-primer2-RNA	CCT CTA ATA CGA CTC ACT ATA GGC GAC CAC TAC CAG CAG AAC A
Primer-EGFP-F490	ACT TCA AGA TCC GCC ACA AC
Primer-EGFP-F473	GAA CGG CAT CAA GGT GAA CT
Primer-EGFP-F536	CGA CCA CTA CCA GCA GAA CA

Activators

Sequence Name	Sequence
Act-GFP-10nt-T-10nt	CCA CTA CCT GAG CAC CCA GTC CGC CCT GAG CAA AGA CCC C
Act-GFP-10nt-NT-10nt	GGG GTC TTT GCT CAG GGC GGA CTG GGT GCT CAG GTA GTG G
Act-GFP-10nt-NT-5MeC-10nt	GGG GTC TTT G/iMe-dC/T CAG GGC GGA CTG GGT GCT CAG GTA GTG G
Act-GFP-10nt-T-5MeC-10nt	CCA CTA CCT GAG CAC CCA GTC CGC CCT GAG /iMe-dC/AA AGA CCC C
Act-GFP-mut-1	CCA CTA CCT GGG CAC CCA GTC CGC CCT GAG CAA AGA CCC C
Act-GFP-mut-2	CCA CTA CCT GAA CAC CCA GTC CGC CCT GAG CAA AGA CCC C
Act-GFP-mut-3	CCA CTA CCT GAG TAC CCA GTC CGC CCT GAG CAA AGA CCC C
Act-GFP-mut-4	CCA CTA CCT GAG CGC CCA GTC CGC CCT GAG CAA AGA CCC C
Act-GFP-mut-5	CCA CTA CCT GAG CAT CCA GTC CGC CCT GAG CAA AGA CCC C
Act-GFP-mut-6	CCA CTA CCT GAG CAC TCA GTC CGC CCT GAG CAA AGA CCC C
Act-GFP-mut-7	CCA CTA CCT GAG CAC CTA GTC CGC CCT GAG CAA AGA CCC C
Act-GFP-mut-8	CCA CTA CCT GAG CAC CCG GTC CGC CCT GAG CAA AGA CCC C
Act-GFP-mut-9	CCA CTA CCT GAG CAC CCA ATC CGC CCT GAG CAA AGA CCC C
Act-GFP-mut-10	CCA CTA CCT GAG CAC CCA GCC CGC CCT GAG CAA AGA CCC C
Act-GFP-mut-11	CCA CTA CCT GAG CAC CCA GTT CGC CCT GAG CAA AGA CCC C
Act-GFP-mut-12	CCA CTA CCT GAG CAC CCA GTC TGC CCT GAG CAA AGA CCC C
Act-GFP-mut-13	CCA CTA CCT GAG CAC CCA GTC CAC CCT GAG CAA AGA CCC C
Act-GFP-mut-14	CCA CTA CCT GAG CAC CCA GTC CGT CCT GAG CAA AGA CCC C
Act-GFP-mut-15	CCA CTA CCT GAG CAC CCA GTC CGC TCT GAG CAA AGA CCC C
Act-GFP-mut-16	CCA CTA CCT GAG CAC CCA GTC CGC CTT GAG CAA AGA CCC C
Act-GFP-mut-17	CCA CTA CCT GAG CAC CCA GTC CGC CCC GAG CAA AGA CCC C
Act-GFP-mut-18	CCA CTA CCT GAG CAC CCA GTC CGC CCT AAG CAA AGA CCC C
Act-GFP-mut-19	CCA CTA CCT GAG CAC CCA GTC CGC CCT GGG CAA AGA CCC C

Act-GFP-mut-20	CCA CTA CCT GAG CAC CCA GTC CGC CCT GAA CAA AGA CCC C
Act-GFP-mut-1 - NT	GGG GTC TTT GCT CAG GGC GGA CTG GGT GCC CAG GTA GTG G
Act-GFP-mut-2 – NT	GGG GTC TTT GCT CAG GGC GGA CTG GGT GTT CAG GTA GTG G
Act-GFP-mut-3 – NT	GGG GTC TTT GCT CAG GGC GGA CTG GGT ACT CAG GTA GTG G
Act-GFP-mut-4 – NT	GGG GTC TTT GCT CAG GGC GGA CTG GGC GCT CAG GTA GTG G
Act-GFP-mut-5 - NT	GGG GTC TTT GCT CAG GGC GGA CTG GAT GCT CAG GTA GTG G
Act-GFP-mut-6 - NT	GGG GTC TTT GCT CAG GGC GGA CTG AGT GCT CAG GTA GTG G
Act-GFP-mut-7 - NT	GGG GTC TTT GCT CAG GGC GGA CTA GGT GCT CAG GTA GTG G
Act-GFP-mut-8 - NT	GGG GTC TTT GCT CAG GGC GGA CCG GGT GCT CAG GTA GTG G
Act-GFP-mut-9 – NT	GGG GTC TTT GCT CAG GGC GGA TTG GGT GCT CAG GTA GTG G
Act-GFP-mut-10 – NT	GGG GTC TTT GCT CAG GGC GGG CTG GGT GCT CAG GTA GTG G
Act-GFP-mut-11 – NT	GGG GTC TTT GCT CAG GGC GAA CTG GGT GCT CAG GTA GTG G
Act-GFP-mut-12 – NT	GGG GTC TTT GCT CAG GGC AGA CTG GGT GCT CAG GTA GTG G
Act-GFP-mut-13 – NT	GGG GTC TTT GCT CAG GGT GGA CTG GGT GCT CAG GTA GTG G
Act-GFP-mut-14 – NT	GGG GTC TTT GCT CAG GAC GGA CTG GGT GCT CAG GTA GTG G
Act-GFP-mut-15 – NT	GGG GTC TTT GCT CAG AGC GGA CTG GGT GCT CAG GTA GTG G
Act-GFP-mut-16 – NT	GGG GTC TTT GCT CAA GGC GGA CTG GGT GCT CAG GTA GTG G
Act-GFP-mut-17 – NT	GGG GTC TTT GCT CGG GGC GGA CTG GGT GCT CAG GTA GTG G
Act-GFP-mut-18 – NT	GGG GTC TTT GCT TAG GGC GGA CTG GGT GCT CAG GTA GTG G
Act-GFP-mut-19 – NT	GGG GTC TTT GCC CAG GGC GGA CTG GGT GCT CAG GTA GTG G
Act-GFP-mut-20 - NT	GGG GTC TTT GTT CAG GGC GGA CTG GGT GCT CAG GTA GTG G
Act-GFP-T-2Xmut1	CCA CTA CCT GGA CAC CCA GTC CGC CCT GAG CAA AGA CCC C
Act-GFP-T-2Xmut2	CCA CTA CCT GAA TAC CCA GTC CGC CCT GAG CAA AGA CCC C
Act-GFP-T-2Xmut3	CCA CTA CCT GAG TGC CCA GTC CGC CCT GAG CAA AGA CCC C
Act-GFP-T-2Xmut4	CCA CTA CCT GAG CGT CCA GTC CGC CCT GAG CAA AGA CCC C

Act-GFP-T-2Xmut5	CCA CTA CCT GAG CAT TCA GTC CGC CCT GAG CAA AGA CCC C
Act-GFP-T-2Xmut6	CCA CTA CCT GAG CAC TTA GTC CGC CCT GAG CAA AGA CCC C
Act-GFP-T-2Xmut7	CCA CTA CCT GAG CAC CTG GTC CGC CCT GAG CAA AGA CCC C
Act-GFP-T-2Xmut8	CCA CTA CCT GAG CAC CCG ATC CGC CCT GAG CAA AGA CCC C
Act-GFP-T-2Xmut9	CCA CTA CCT GAG CAC CCA ACC CGC CCT GAG CAA AGA CCC C
Act-GFP-T-2Xmut10	CCA CTA CCT GAG CAC CCA GCT CGC CCT GAG CAA AGA CCC C
Act-GFP-T-2Xmut11	CCA CTA CCT GAG CAC CCA GTT TGC CCT GAG CAA AGA CCC C
Act-GFP-T-2Xmut12	CCA CTA CCT GAG CAC CCA GTC TAC CCT GAG CAA AGA CCC C
Act-GFP-T-2Xmut13	CCA CTA CCT GAG CAC CCA GTC CAT CCT GAG CAA AGA CCC C
Act-GFP-T-2Xmut14	CCA CTA CCT GAG CAC CCA GTC CGT TCT GAG CAA AGA CCC C
Act-GFP-T-2Xmut15	CCA CTA CCT GAG CAC CCA GTC CGC TTT GAG CAA AGA CCC C
Act-GFP-T-2Xmut16	CCA CTA CCT GAG CAC CCA GTC CGC CTC GAG CAA AGA CCC C
Act-GFP-T-2Xmut17	CCA CTA CCT GAG CAC CCA GTC CGC CCC AAG CAA AGA CCC C
Act-GFP-T-2Xmut18	CCA CTA CCT GAG CAC CCA GTC CGC CCT AGG CAA AGA CCC C
Act-GFP-T-2Xmut19	CCA CTA CCT GAG CAC CCA GTC CGC CCT GGA CAA AGA CCC C
Act-GFP-NT-2Xmut1	GGG GTC TTT GCT CAG GGC GGA CTG GGT GTC CAG GTA GTG G
Act-GFP-NT-2Xmut2	GGG GTC TTT GCT CAG GGC GGA CTG GGT ATT CAG GTA GTG G
Act-GFP-NT-2Xmut3	GGG GTC TTT GCT CAG GGC GGA CTG GGC ACT CAG GTA GTG G
Act-GFP-NT-2Xmut4	GGG GTC TTT GCT CAG GGC GGA CTG GAC GCT CAG GTA GTG G
Act-GFP-NT-2Xmut5	GGG GTC TTT GCT CAG GGC GGA CTG AAT GCT CAG GTA GTG G
Act-GFP-NT-2Xmut6	GGG GTC TTT GCT CAG GGC GGA CTA AGT GCT CAG GTA GTG G
Act-GFP-NT-2Xmut7	GGG GTC TTT GCT CAG GGC GGA CCA GGT GCT CAG GTA GTG G
Act-GFP-NT-2Xmut8	GGG GTC TTT GCT CAG GGC GGA TCG GGT GCT CAG GTA GTG G
Act-GFP-NT-2Xmut9	GGG GTC TTT GCT CAG GGC GGG TTG GGT GCT CAG GTA GTG G
Act-GFP-NT-2Xmut10	GGG GTC TTT GCT CAG GGC GAG CTG GGT GCT CAG GTA GTG G

Act-GFP-NT-2Xmut11	GGG GTC TTT GCT CAG GGC AAA CTG GGT GCT CAG GTA GTG G
Act-GFP-NT-2Xmut12	GGG GTC TTT GCT CAG GGT AGA CTG GGT GCT CAG GTA GTG G
Act-GFP-NT-2Xmut13	GGG GTC TTT GCT CAG GAT GGA CTG GGT GCT CAG GTA GTG G
Act-GFP-NT-2Xmut14	GGG GTC TTT GCT CAG AAC GGA CTG GGT GCT CAG GTA GTG G
Act-GFP-NT-2Xmut15	GGG GTC TTT GCT CAA AGC GGA CTG GGT GCT CAG GTA GTG G
Act-GFP-NT-2Xmut16	GGG GTC TTT GCT CGA GGC GGA CTG GGT GCT CAG GTA GTG G
Act-GFP-NT-2Xmut17	GGG GTC TTT GCT TGG GGC GGA CTG GGT GCT CAG GTA GTG G
Act-GFP-NT-2Xmut18	GGG GTC TTT GCC TAG GGC GGA CTG GGT GCT CAG GTA GTG G
Act-GFP-NT-2Xmut19	GGG GTC TTT GTC CAG GGC GGA CTG GGT GCT CAG GTA GTG G
CoV2-Act-T-WT	GGT TAC TGC CAG TTG AAT CTG AGG GTC CAC CAA ACG TAA T
CoV2-Act-T-mut1	GGT TAC TGC CGG TTG AAT CTG AGG GTC CAC CAA ACG TAA T
CoV2-Act-T-mut2	GGT TAC TGC CAA TTG AAT CTG AGG GTC CAC CAA ACG TAA T
CoV2-Act-T-mut3	GGT TAC TGC CAG CTG AAT CTG AGG GTC CAC CAA ACG TAA T
CoV2-Act-T-mut4	GGT TAC TGC CAG TCG AAT CTG AGG GTC CAC CAA ACG TAA T
CoV2-Act-T-mut5	GGT TAC TGC CAG TTA AAT CTG AGG GTC CAC CAA ACG TAA T
CoV2-Act-T-mut6	GGT TAC TGC CAG TTG GAT CTG AGG GTC CAC CAA ACG TAA T
CoV2-Act-T-mut7	GGT TAC TGC CAG TTG AGT CTG AGG GTC CAC CAA ACG TAA T
CoV2-Act-T-mut8	GGT TAC TGC CAG TTG AAC CTG AGG GTC CAC CAA ACG TAA T
CoV2-Act-T-mut9	GGT TAC TGC CAG TTG AAT TTG AGG GTC CAC CAA ACG TAA T
CoV2-Act-T-mut10	GGT TAC TGC CAG TTG AAT CCG AGG GTC CAC CAA ACG TAA T
CoV2-Act-T-mut11	GGT TAC TGC CAG TTG AAT CTA AGG GTC CAC CAA ACG TAA T
CoV2-Act-T-mut12	GGT TAC TGC CAG TTG AAT CTG GGG GTC CAC CAA ACG TAA T
CoV2-Act-T-mut13	GGT TAC TGC CAG TTG AAT CTG AAG GTC CAC CAA ACG TAA T
CoV2-Act-T-mut14	GGT TAC TGC CAG TTG AAT CTG AGA GTC CAC CAA ACG TAA T
CoV2-Act-T-mut15	GGT TAC TGC CAG TTG AAT CTG AGG ATC CAC CAA ACG TAA T

CoV2-Act-T-mut16	GGT TAC TGC CAG TTG AAT CTG AGG GCC CAC CAA ACG TAA T
CoV2-Act-T-mut17	GGT TAC TGC CAG TTG AAT CTG AGG GTT CAC CAA ACG TAA T
CoV2-Act-T-mut18	GGT TAC TGC CAG TTG AAT CTG AGG GTC TAC CAA ACG TAA T
CoV2-Act-T-mut19	GGT TAC TGC CAG TTG AAT CTG AGG GTC CGC CAA ACG TAA T
CoV2-Act-T-mut20	GGT TAC TGC CAG TTG AAT CTG AGG GTC CAT CAA ACG TAA T
CoV2-Act-NT-WT	ATT ACG TTT GGT GGA CCC TCA GAT TCA ACT GGC AGT AAC C
CoV2-Act-NT-mut1	ATT ACG TTT GGT GGA CCC TCA GAT TCA ACC GGC AGT AAC C
CoV2-Act-NT-mut2	ATT ACG TTT GGT GGA CCC TCA GAT TCA ATT GGC AGT AAC C
CoV2-Act-NT-mut3	ATT ACG TTT GGT GGA CCC TCA GAT TCA GCT GGC AGT AAC C
CoV2-Act-NT-mut4	ATT ACG TTT GGT GGA CCC TCA GAT TCG ACT GGC AGT AAC C
CoV2-Act-NT-mut5	ATT ACG TTT GGT GGA CCC TCA GAT TTA ACT GGC AGT AAC C
CoV2-Act-NT-mut6	ATT ACG TTT GGT GGA CCC TCA GAT CCA ACT GGC AGT AAC C
CoV2-Act-NT-mut7	ATT ACG TTT GGT GGA CCC TCA GAC TCA ACT GGC AGT AAC C
CoV2-Act-NT-mut8	ATT ACG TTT GGT GGA CCC TCA GGT TCA ACT GGC AGT AAC C
CoV2-Act-NT-mut9	ATT ACG TTT GGT GGA CCC TCA AAT TCA ACT GGC AGT AAC C
CoV2-Act-NT-mut10	ATT ACG TTT GGT GGA CCC TCG GAT TCA ACT GGC AGT AAC C
CoV2-Act-NT-mut11	ATT ACG TTT GGT GGA CCC TTA GAT TCA ACT GGC AGT AAC C
CoV2-Act-NT-mut12	ATT ACG TTT GGT GGA CCC CCA GAT TCA ACT GGC AGT AAC C
CoV2-Act-NT-mut13	ATT ACG TTT GGT GGA CCT TCA GAT TCA ACT GGC AGT AAC C
CoV2-Act-NT-mut14	ATT ACG TTT GGT GGA CTC TCA GAT TCA ACT GGC AGT AAC C
CoV2-Act-NT-mut15	ATT ACG TTT GGT GGA TCC TCA GAT TCA ACT GGC AGT AAC C
CoV2-Act-NT-mut16	ATT ACG TTT GGT GGG CCC TCA GAT TCA ACT GGC AGT AAC C
CoV2-Act-NT-mut17	ATT ACG TTT GGT GAA CCC TCA GAT TCA ACT GGC AGT AAC C
CoV2-Act-NT-mut18	ATT ACG TTT GGT AGA CCC TCA GAT TCA ACT GGC AGT AAC C
CoV2-Act-NT-mut19	ATT ACG TTT GGC GGA CCC TCA GAT TCA ACT GGC AGT AAC C

CoV2-Act-NT-mut20	ATT ACG TTT GAT GGA CCC TCA GAT TCA ACT GGC AGT AAC C
CoV2-Act-T-2Xmut1	GGT TAC TGC CGA TTG AAT CTG AGG GTC CAC CAA ACG TAA T
CoV2-Act-T-2Xmut2	GGT TAC TGC CAA CTG AAT CTG AGG GTC CAC CAA ACG TAA T
CoV2-Act-T-2Xmut3	GGT TAC TGC CAG CCG AAT CTG AGG GTC CAC CAA ACG TAA T
CoV2-Act-T-2Xmut4	GGT TAC TGC CAG TCA AAT CTG AGG GTC CAC CAA ACG TAA T
CoV2-Act-T-2Xmut5	GGT TAC TGC CAG TTA GAT CTG AGG GTC CAC CAA ACG TAA T
CoV2-Act-T-2Xmut6	GGT TAC TGC CAG TTG GGT CTG AGG GTC CAC CAA ACG TAA T
CoV2-Act-T-2Xmut7	GGT TAC TGC CAG TTG AGC CTG AGG GTC CAC CAA ACG TAA T
CoV2-Act-T-2Xmut8	GGT TAC TGC CAG TTG AAC TTG AGG GTC CAC CAA ACG TAA T
CoV2-Act-T-2Xmut9	GGT TAC TGC CAG TTG AAT TCG AGG GTC CAC CAA ACG TAA T
CoV2-Act-T-2Xmut10	GGT TAC TGC CAG TTG AAT CCA AGG GTC CAC CAA ACG TAA T
CoV2-Act-T-2Xmut11	GGT TAC TGC CAG TTG AAT CTA GGG GTC CAC CAA ACG TAA T
CoV2-Act-T-2Xmut12	GGT TAC TGC CAG TTG AAT CTG GAG GTC CAC CAA ACG TAA T
CoV2-Act-T-2Xmut13	GGT TAC TGC CAG TTG AAT CTG AAA GTC CAC CAA ACG TAA T
CoV2-Act-T-2Xmut14	GGT TAC TGC CAG TTG AAT CTG AGA ATC CAC CAA ACG TAA T
CoV2-Act-T-2Xmut15	GGT TAC TGC CAG TTG AAT CTG AGG ACC CAC CAA ACG TAA T
CoV2-Act-T-2Xmut16	GGT TAC TGC CAG TTG AAT CTG AGG GCT CAC CAA ACG TAA T
CoV2-Act-T-2Xmut17	GGT TAC TGC CAG TTG AAT CTG AGG GTT TAC CAA ACG TAA T
CoV2-Act-T-2Xmut18	GGT TAC TGC CAG TTG AAT CTG AGG GTC TGC CAA ACG TAA T
CoV2-Act-T-2Xmut19	GGT TAC TGC CAG TTG AAT CTG AGG GTC CGT CAA ACG TAA T
CoV2-Act-NT-2Xmut1	ATT ACG TTT GGT GGA CCC TCA GAT TCA ATC GGC AGT AAC C
CoV2-Act-NT-2Xmut2	ATT ACG TTT GGT GGA CCC TCA GAT TCA GTT GGC AGT AAC C
CoV2-Act-NT-2Xmut3	ATT ACG TTT GGT GGA CCC TCA GAT TCG GCT GGC AGT AAC C
CoV2-Act-NT-2Xmut4	ATT ACG TTT GGT GGA CCC TCA GAT TTG ACT GGC AGT AAC C
CoV2-Act-NT-2Xmut5	ATT ACG TTT GGT GGA CCC TCA GAT CTA ACT GGC AGT AAC C

CoV2-Act-NT-2Xmut6	ATT ACG TTT GGT GGA CCC TCA GAC CCA ACT GGC AGT AAC C
CoV2-Act-NT-2Xmut7	ATT ACG TTT GGT GGA CCC TCA GGC TCA ACT GGC AGT AAC C
CoV2-Act-NT-2Xmut8	ATT ACG TTT GGT GGA CCC TCA AGT TCA ACT GGC AGT AAC C
CoV2-Act-NT-2Xmut9	ATT ACG TTT GGT GGA CCC TCG AAT TCA ACT GGC AGT AAC C
CoV2-Act-NT-2Xmut10	ATT ACG TTT GGT GGA CCC TTG GAT TCA ACT GGC AGT AAC C
CoV2-Act-NT-2Xmut11	ATT ACG TTT GGT GGA CCC CTA GAT TCA ACT GGC AGT AAC C
CoV2-Act-NT-2Xmut12	ATT ACG TTT GGT GGA CCT CCA GAT TCA ACT GGC AGT AAC C
CoV2-Act-NT-2Xmut13	ATT ACG TTT GGT GGA CTT TCA GAT TCA ACT GGC AGT AAC C
CoV2-Act-NT-2Xmut14	ATT ACG TTT GGT GGA TTC TCA GAT TCA ACT GGC AGT AAC C
CoV2-Act-NT-2Xmut15	ATT ACG TTT GGT GGG TCC TCA GAT TCA ACT GGC AGT AAC C
CoV2-Act-NT-2Xmut16	ATT ACG TTT GGT GAG CCC TCA GAT TCA ACT GGC AGT AAC C
CoV2-Act-NT-2Xmut17	ATT ACG TTT GGT AAA CCC TCA GAT TCA ACT GGC AGT AAC C
CoV2-Act-NT-2Xmut18	ATT ACG TTT GGC AGA CCC TCA GAT TCA ACT GGC AGT AAC C
CoV2-Act-NT-2Xmut19	ATT ACG TTT GAC GGA CCC TCA GAT TCA ACT GGC AGT AAC C
